# Basis Set Incompleteness Errors in Fixed-Node Diffusion Monte Carlo Calculations on Noncovalent Interactions

**DOI:** 10.1021/acs.jctc.4c01631

**Published:** 2025-04-30

**Authors:** Kousuke Nakano, Benjamin X. Shi, Dario Alfè, Andrea Zen

**Affiliations:** † Center for Basic Research on Materials, National Institute for Materials Science (NIMS), Tsukuba, Ibaraki 305-0047, Japan; ‡ Yusuf Hamied Department of Chemistry, University of Cambridge, Cambridge CB2 1EW, United Kingdom; § Dipartimento di Fisica Ettore Pancini, Università di Napoli Federico II, Monte S. Angelo, I-80126 Napoli, Italy; ∥ Department of Earth Sciences, University College London, Gower Street, London WC1E 6BT, United Kingdom; ⊥ Thomas Young Centre and London Centre for Nanotechnology, 17-19 Gordon Street, London WC1H 0AH, United Kingdom

## Abstract

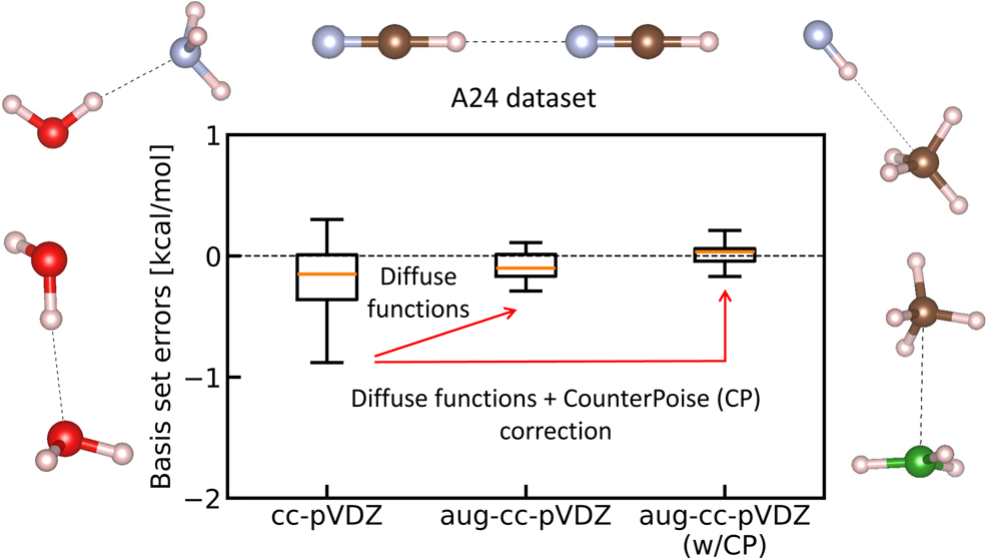

Basis set incompleteness error (BSIE) is a common source of error in quantum chemistry calculations, but it has not been comprehensively studied in fixed-node Diffusion Monte Carlo (FN-DMC) calculations. FN-DMC, being a projection method, is often considered minimally affected by basis set biases. Here, we show that this assumption is not always valid. While the relative error introduced by a small basis set in the total FN-DMC energy is minor, it can become significant in binding energy (*E*_b_) evaluations of weakly interacting systems. We systematically investigated BSIEs in FN-DMC-based *E*_b_ evaluations using the A24 data set, a well-known benchmark set of 24 noncovalently bound dimers. We found that BSIEs in FN-DMC evaluations of *E*_b_ are indeed significant when small localized basis sets, such as cc-pVDZ and cc-pVTZ, are employed. Our study shows that the aug-cc-pVTZ basis set family strikes a good balance between computational cost and BSIEs in the *E*_b_ calculations. We also found that augmenting the basis sets with diffuse orbitals, using counterpoise correction, or both, effectively mitigates BSIEs, allowing smaller basis sets such as aug-cc-pVDZ to be used.

## 1 Introduction

Diffusion Monte Carlo (DMC)^1,2^ is a state-of-the-art electronic structure method used for predicting and understanding phenomena in materials science, chemistry, and physics. In particular, DMC can achieve highly accurate quantitative predictions, typically surpassing those of mean-field approaches like density functional theory (DFT). This level of accuracy has proven essential for studying systems challenging for DFT, such as high-pressure hydrogen,^3−9^ layered materials,^10−14^ molecular crystals,^15,16^ and molecular adsorption on surfaces.^17−21^

In theory, DMC is an exact technique to project the ground state (GS) of a Hamiltonian. However, in practical applications to Fermionic systems (e.g., atoms, molecules, and materials), it relies on the fixed-node (FN) approximation to maintain the antisymmetry of the wave function. The FN approximation constrains the nodal surface of the projected state to that of a trial wave function, which can be generated by methods such as DFT, Hartree–Fock (HF), or correlated quantum chemistry (QC) methods, including the complete active space self-consistent field (CASSCF) method.

The approaches used to generate the trial wave function are not exact, so its nodal surface is not exact either, yielding an error on the FN-DMC evaluations called the FN error. The closer the nodal surface of the trial wave function to the nodal surface of the exact GS, the smaller the FN error. There are other approximations in FN-DMC, but typically the major source of error is the FN error. The FN error depends on the accuracy of the trial wave function, which in turn depends on the level of theory employed to generate it (e.g., we expect a CASSCF wave function to have a better nodal surface than a DFT or a HF wave function) and on the completeness of the employed basis set representation. In general, a larger basis set gives a better wave function (i.e., nodal surface), although a few exceptions are reported.^22^ One typically chooses a basis set by considering the trade-off between costs (i.e., CPU time + memory requirement) and accuracy. On the one hand, using a too large basis set increases the computational cost and the memory requirement with little benefit. This issue becomes particularly prominent when constructing a multideterminant trial wave function^23^ or generating a single-determinant trial wave function with methods beyond mean-field theory.^24^ Specifically, several of the authors have recently proposed a way to generate a trial wave function based on the natural orbitals constructed from a second-order Møller–Plesset (MP2) calculation,^24^ allowing one to go beyond the single-reference fixed-node approximation. However, the workflow becomes impractical for large molecules in terms of memory and storage when the basis set size is increased, due to the steep scaling of MP2. On the other hand, using a too small basis set risks introducing bias into FN-DMC results. The choice of a basis set family balancing the accuracy and the computational cost is also particularly pertinent for a calculation spanning chemical space, such as developing machine-learning models.^25^ While previous works have explored the influence of trial wave function accuracy,^26−32^ the impact of the basis set incompleteness errors (BSIEs) in FN-DMC has yet to be comprehensively and systematically explored in the context of noncovalent interaction evaluations, which is one of the most prominent applications of FN-DMC.^10−14,15,21,27,33−40^

In QC and DFT methods, BSIEs are a dominant error source that requires careful control, yet it has been often assumed that FN-DMC is relatively immune to BSIEs from the trial wave function^41^ because it depends only on the nodal surface, not on the full wave function amplitude. In this work, we systematically investigate how these assumptions hold up by analyzing BSIEs in FN-DMC calculations.

BSIEs are especially pronounced in QC and DFT methods when describing noncovalent interactions. In this context, the quantity of interest is typically the binding energy of a dimer complex (AB), defined as
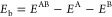
1where *E*^A^, *E*^B^, and *E*^AB^ are the total energies of monomer A, monomer B, and the AB dimer complex, respectively. This study focuses on the propagation of BSIEs from the trial wave function in FN-DMC calculations of *E*_b_, a particularly relevant area of investigation given the high sensitivity of noncovalent interactions to basis set quality.^24,39,42^

A basis set consists of a number of basis functions that are used to represent the electronic wave function, with the complete basis set (CBS) limit achieved when expanded toward an (infinite) set of functions. The BSIE is the deviation from the CBS limit^43,44^ and for a binding energy *E*_b_, it is defined as^43,44^
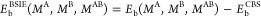
2where *M*^A^, *M*^B^ and *M*^AB^ denote the number and type of basis functions employed in the calculation of *E*^A^, *E*^B^ and *E*^AB^ respectively within eq 1, and *E*_b_^CBS^ denotes the binding energy in the CBS limit. Two common choices of basis function types are plane waves (PWs) and atom-centered Gaussian Type Orbitals (GTOs). On the one hand, BSIEs are well-controlled with PWs because systematic convergence toward the CBS limit can be achieved by monotonically increasing the kinetic (i.e., cutoff) energy of the included PWs. On the other hand, errors in GTOs are less well-behaved, with users selecting from ‘families’ of available basis sets consisting of increasing sizes, often denoted by the number of ‘zeta’ basis functions per occupied valence orbital. A popular example is the correlation consistent basis-set family, developed by Dunning and co-workers,^45^ for instance the correlation-consistent polarized valence *n*-zeta (cc-pV*n*Z), where *n*, the cardinal number, can take on double (D), triple (T), quadruple (Q), quintuple (5) and sextuple (6) zeta functions on each atom. It is also common to augment these with additional diffuse functions, which are denoted by an ‘aug-’ prefix in front.

When using GTOs, or any other set of atom-centered basis functions, to compute binding energies, it is crucial to distinguish BSIEs from basis-set superposition errors (BSSEs),^43,44^ a related source of error. BSSE occurs when basis functions of interacting molecular systems A and B in the AB dimer overlap, increasing the variational space for the AB dimer with respect to the A and B monomers, thus leading to an overestimation of *E*_b_.^1^ This error is defined by Boys and Bernardi^46^ as
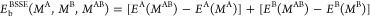
3involving two separate calculations on each monomer. For monomer A, alongside the original basis set *E*^A^(*M*^A^), a calculation including additional empty ‘ghost’ functions from monomer B is also performed to get *E*^A^(*M*^AB^), as proposed by Boys and Bernardi.^46^ The difference between the two quantities, appearing in eq 3, then provides an estimate on the effect of the basis set superposition on the energy of each monomer. Thus, the BSSE error *E*_b_^BSSE^ can be used to correct the original *E*_b_ evaluation to obtain a counterpoise (CP) corrected estimate of the binding energy: *E*_b_^CP^ = *E*_b_ – *E*_b_^BSSE^. It must be emphasized that the CP corrected estimates still suffer from BSIE, although they are typically closer to the CBS limit,^44^ and typically underbind *E*_b_.^2^ In the CBS limit, both BSIE and BSSE will vanish.

To date, only a few studies have reported BSIEs in FN-DMC for *E*_b_ calculations of noncovalent interactions and to our knowledge, none have studied the effect of CP corrections. Korth et al.^26^ reported the difference between noncovalent interaction energies of the Li-thiophene complex obtained with cc-pVTZ and cc-pVQZ basis sets. The results from the cc-pVQZ basis were close to the CCSD(T)/CBS reference value. Dubecký et al.^34^ studied the effect of the cardinal number *n* and augmentation functions in ammonia dimer. On the one hand, they revealed that the higher cardinality number *n* (from cc-pVTZ to cc-pVQZ) has a smaller effect on the overall accuracy than the augmentation does. On the other hand, the additional diffuse functions (aug-) were found to be crucial to reach the reference CCSD(T)/CBS interaction energy value because the augmentation functions likely improve the tails of trial wave functions that are crucial for describing van der Waals complexes correctly. They recommended the aug-cc-pVTZ basis set as the most reasonable choice with respect to the price/performance ratio. Very recently, Zhou et al.^47^ evaluated barrier heights and complexation energies in small water, ammonia, and hydrogen fluoride clusters using FN-DMC with basis sets of increasing completeness, and recommend basis sets containing diffuse basis functions.

In this paper, we present a detailed analysis of the basis set effects, BSIEs and BSSEs, in DMC binding energy calculations, specifically focusing on noncovalent interactions. Our findings indicate that while BSIEs and BSSEs in FN-DMC are substantially reduced compared to those in the trial wave function, they are not negligible. The key conclusions to get CBS-limit binding energies (i.e., negligible BSIEs and BSSEs) from our work are (1) aug-cc-pVDZ is sufficient when CP correction is applied and (2) the aug-cc-pVTZ basis set performs well without the need for CP correction.

## 2 Computational Details

To investigate BSIEs in DMC calculations systematically, we computed binding energies (*E*_b_) of the complex systems included in the A24 data set.^48^ The A24 data set is a set of noncovalently bound dimers, consisting of systems dominated by H-bonding, dispersion and a mixture of both.^48^ The data set was intended to test the accuracy of computational methods that are used as benchmarks in larger model systems. We employed the correlation consistent (cc) GTOs accompanied by the correlation consistent effective core potentials^49,50^ (ccECP) in this study. The majority of the QMC results reported in this work are obtained using the TurboRVB(51) ab initio QMC packages. TurboRVB performs QMC calculations using trial wave functions expressed in terms of localized atomic orbitals, such as GTOs. TurboRVB supports the CP correction for QMC calculations using trial wave functions with GTOs, allowing one to study both BSIEs and BSSEs. More specifically, TurboRVB can assign GTOs to the so-called ghost atoms (i.e., with zero nuclear charges), as in QC calculations.

TurboRVB implements the lattice discretized version of the FN-DMC calculations (LRDMC).^51,52^ Notice that the infinitesimal mesh limit of LRDMC evaluations is equivalent to the infinitesimal time step limit in standard DMC evaluations, provided that the computational setup (i.e., trial wave function, pseudopotential, localization approximation of the nonlocal pseudopotential terms) is the same. The LRDMC calculations with TurboRVB were performed by the single-grid scheme^52^ with lattice spaces *a* = 0.30, 0.25, 0.20, and 0.10 Bohr. BSSEs were computed at each lattice space according to eq 3, and then the obtained values were extrapolated to *a* → 0 using *E*_b_^BSSE^(*a*^2^) = *k*_2_ · *a*^2^ + *E*_b_^BSSE^, where *E*_b_^BSSE^ is the extrapolated BSSE. In computing BSIEs, the binding energies computed with the aug-cc-pV6Z were used as the reference values, i.e., *E*_b_^CBS^ in eq 2, for each complex system because, as shown in the following section, the aug-cc-pV6Z basis has reached the CBS limit. The binding energy obtained with each basis set was extrapolated to *a* → 0 using *E*_b_(*a*^2^) = *k*_4_ · *a*^4^ + *k*_2_ · *a*^2^ + *E*_b_, where *E*_b_ is the extrapolated binding energy, and then BSIEs were computed according to eq 2. While the wave function variances differ across the various basis sets, this does not affect the LRDMC extrapolations because we reduced the error bars for each lattice space to the same value in all basis sets (See Table SII and Figure S2 of the SI).

The ccECP pseudopotentials are semilocal effective core potentials, as with most available pseudopotentials, so the DMC results depend on how the sign problem from its nonlocal term is addressed. In this study, we used the determinant locality T-move (DTM)^53^ scheme in the majority of the calculations shown here, which are performed with TurboRVB.

For the DFT calculations that generate trial wave functions with GTOs for subsequent QMC calculations via TREX-IO(54) files, we used the PySCF(55,56) package, with the PZ-LDA^57^ exchange-correlation functional. For LRDMC calculations with TurboRVB the obtained trial wave functions are combined with the two-body and the three-body Jastrow factors.^51^ The three-body Jastrow factors are not attached to the ghost atoms in CP calculations. The parameters in the Jastrow factors were optimized using the Stochastic Reconfiguration method.^58^ We notice that the optimization of the Jastrow factor does not affect the extrapolated LRDMC total and binding energies since the DTM is employed in this study. In this sense, the obtained conclusions in this study are deterministic.

We also compare QMC evaluations obtained using PW basis sets in comparison with localized GTO basis sets (Table SI of the Supporting Information (SI)). The comparison uses results obtained with the QMCPACK package,^59,60^ which implements wave functions using either PW or GTO basis sets. Details about the QMCPACK calculations are provided in Section 1 of the SI.

## 3 Basis-Set Convergence Checks to Estimate the Binding Energies in the CBS Limit

To estimate BSIEs, the binding energies in the CBS limit are needed, as described in eq 2. Since zero BSSE implies zero BSIE in binding energy calculation, computing BSSEs is helpful to decide which basis set should be used to compute *E*_b_^CBS^ in eq 2.

**Figure 1:**
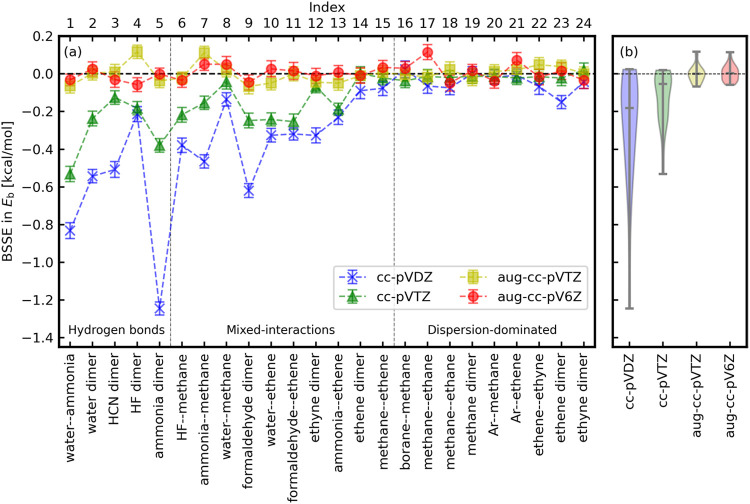
(a) The BSSEs in the binding energies of the A24 set computed by LRDMC with cc-pVDZ, cc-pVTZ, aug-cc-pVTZ, and aug-cc-pV6Z basis sets. The plotted BSSEs are the values extrapolated to the infinitesimal lattice space. The error bars represent 1σ. (b) The violin plots for the obtained BSSEs, with median of the distribution indicated with a gray line inside the violin plot.

Figure 1(a) shows BSSEs in the binding energies of the A24 set computed by LRDMC implemented in TurboRVB. They were obtained using cc-pVDZ, cc-pVTZ, aug-cc-pVTZ, and aug-cc-pV6Z basis sets. Figure 1 (b) shows the violin plots of the BSSEs. The figures reveal that the binding energies obtained with the cc-pVDZ and cc-pVTZ basis sets have significant BSSEs, indicating that the small basis sets are far from the CBS limit. BSSEs vanish for all molecules with the aug-cc-pV6Z basis set within an interval of three standard deviations (±3σ, corresponding to a confidence of 99.7%), indicating that the aug-cc-pV6Z basis set has reached the CBS limit.

In addition, to double-check that the aug-cc-pV6Z basis set gives the CBS-limit binding energies, we computed the DMC binding energies on all A24 dimers using aug-cc-pV6Z, as well as smaller GTO basis sets, and PW basis sets with a very large cutoff. We made this calculations using QMCPACK, which allows to use both localized and PW basis sets. The binding energies values are reported in Table SI of the SI, and a comparison between the evaluations with different basis sets is shown in Figure S1 of the SI. The results indicate that aug-cc-pV6Z and large-cutoff-PW basis sets give consistent binding energies within an interval of ±3σ, supporting the above argument that the aug-cc-pV6Z basis set gives converged binding energies.

Therefore, both the BSSEs evaluation and the comparison with large-cutoff-PW indicate that the aug-cc-pV6Z has reached the CBS limit. Thus, we can use the binding energies obtained with the aug-cc-pV6Z basis sets (without the CP correction) as reference values (i.e., *E*_b_^CBS^ in eq 2) in the following BSIE analysis. The reference DMC values are reported in Table 1.

**Table 1:** Binding Energies *E*_b_, in kcal/mol, of the 24 Molecular Dimers Contained in the A24 Dataset^48^^,^^a^

label	*E*_b_^DMC^	*E*_b_^CCSD(T)^	Δ
water--ammonia	–6.75(7)	–6.493	0.26(7)
water dimer	–5.10(8)	–5.006	0.09(8)
HCN dimer	–5.09(7)	–4.745	0.34(7)
HF dimer	–4.74(7)	–4.581	0.16(7)
ammonia dimer	–3.10(6)	–3.137	–0.04(6)
HF--methane	–1.64(7)	–1.654	–0.01(7)
ammonia--methane	–0.80(7)	–0.765	0.04(7)
water--methane	–0.58(6)	–0.663	–0.08(6)
formaldehyde dimer	–4.42(9)	–4.554	–0.13(9)
water--ethene	–2.50(10)	–2.557	–0.06(10)
formaldehyde--ethene	–1.71(10)	–1.621	0.09(10)
ethyne dimer	–1.44(7)	–1.524	–0.08(7)
ammonia--ethene	–1.38(6)	–1.374	0.01(6)
ethene dimer	–0.97(9)	–1.090	–0.12(9)
methane--ethene	–0.56(6)	–0.502	0.06(6)
borane--methane	–1.46(7)	–1.485	–0.03(7)
methane--ethane	–0.65(9)	–0.827	–0.18(9)
methane--ethane	–0.57(8)	–0.607	–0.04(8)
methane dimer	–0.58(6)	–0.533	0.05(6)
Ar--methane	–0.36(8)	–0.405	–0.05(8)
Ar--ethene	–0.24(7)	–0.364	–0.12(7)
ethene--ethyne	1.04(9)	0.821	–0.22(9)
ethene dimer	1.04(8)	0.934	–0.11(8)
ethyne dimer	1.32(8)	1.115	–0.21(8)
RMSD	—	—	0.135

a *E*_b_^DMC^ column shows results obtained in this work, from LRDMC calculations employing the ccECP pseudopotentials^49,50^ with the DTM approximation,^53^ and a trial wavefunction with the determinant from a LDA-PZ DFT calculation, constructed with ccecp-aug-cc-pV6Z basis sets. *E*_b_^CCSD(T)^ column shows the evaluations from Řezáč and Hobza,^48^ computed by CCSD(T) with extrapolations to the CBS limits. The last column shows the differences Δ = *E*_b_^CCSD(T)^ – *E*_b_^DMC^ between the LRDMC and CCSD(T) values, with the root mean square deviation RMSD at the end.

Additionally, for the ammonia dimer, we also tested trial wave functions with the following XC functionals: PBE,^61^ PBE0,^62^ B3LYP,^63^ ωB97M-V^64^ and Hartree–Fock, to determine whether the convergence behavior depends on the choice of XC, as shown in Table SV and SVI of the SI. The results demonstrate that neither the binding energy nor the convergence behavior depends on the XC functional employed.

## 4 Bias against the Binding Energies in the CBS Limit

**Figure 2:**
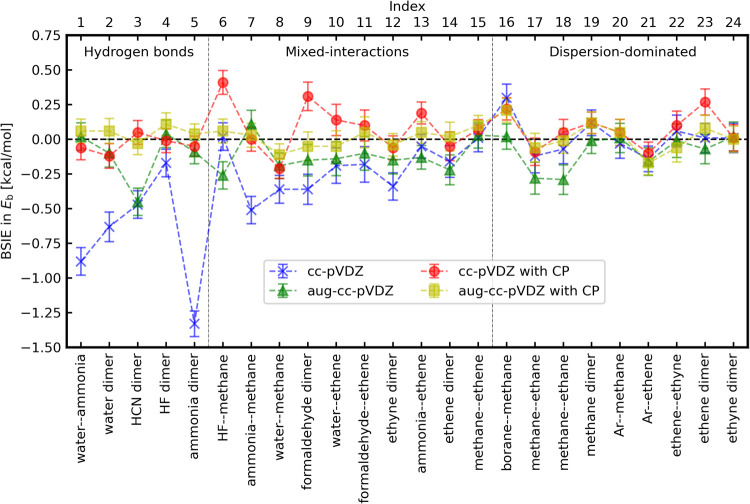
BSIEs in the binding energies of the A24 set, estimated from LRDMC calculations in the limit of infinitesimal lattice spaces. The error bars represent 1σ.

**Figure 3:**
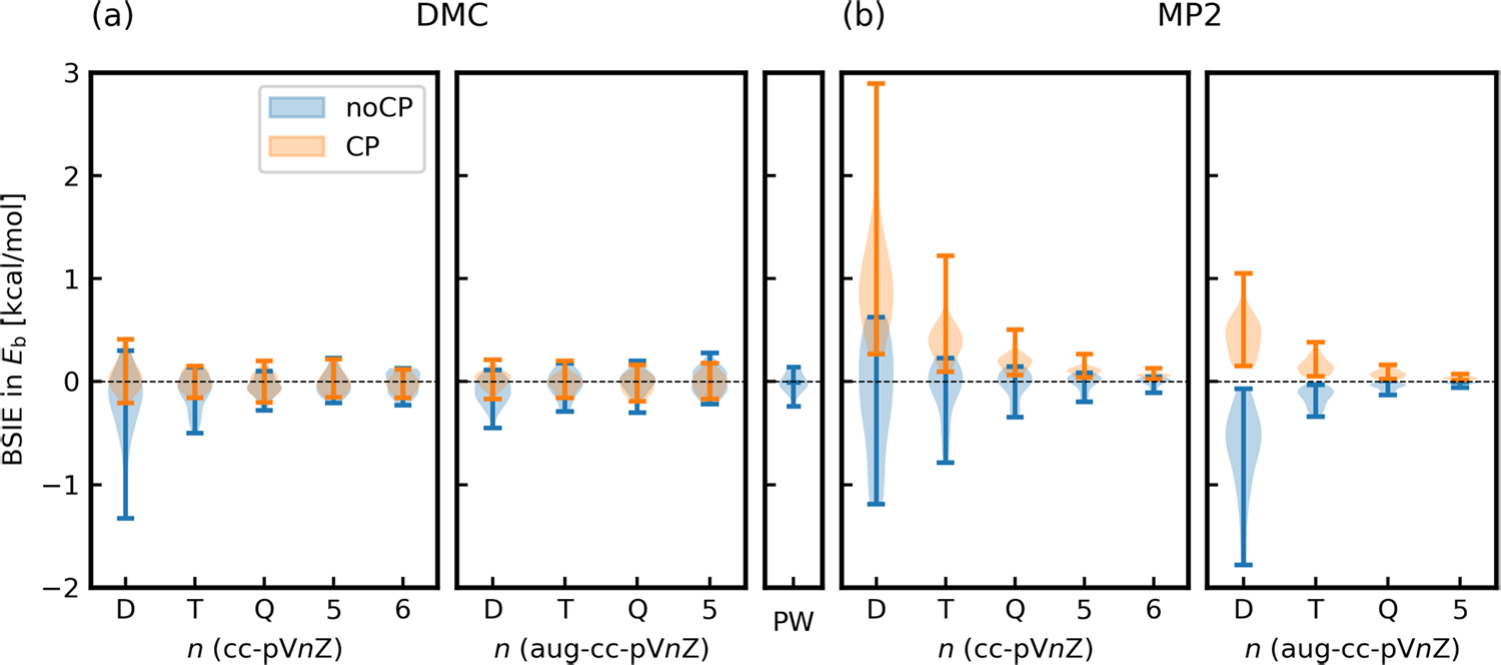
Violin plots of BSIEs in the binding energy calculations of the A24 data set with and without the CP corrections. (a) LRDMC with cc-pV*n*Z (*n* = D,T,Q,5,6) and aug-cc-pV*n*Z (*n* = D,T,Q,5) and DMC with PW. (b) MP2 with cc-pV*n*Z (*n* = D,T,Q,5,6) and with aug-cc-pV*n*Z (*n* = D,T,Q,5). The reference binding energies are those obtained with aug-cc-pV6Z basis without CP correction.

BSIEs in binding energies obtained from LRDMC calculations with the cc-pVDZ and aug-cc-pVDZ basis sets (for the ccECPs^49,50^) with and without the CP corrections are shown in Figure 2 for each of the 24 dimers of the A24 data set. The distribution of the BSIEs across the data set for the same basis set and CP correction combinations is shown in Figure 3(a) via a violin plot. By comparison, BSIEs in MP2 calculations are shown in Figure 3(b).

In Figure 2, the comparison between the BSIEs with cc-pVDZ (without CP) and with aug-cc-pVDZ (without CP) reveals that the augmentation of the basis set drastically decreases BSIEs, specifically for the complex systems with hydrogen-bond interactions. The most significant discrepancy is seen for the ammonia dimer, for which Dubecký et al.^34^ also reported that the additional diffuse functions (i.e., augmentation) were crucial to reach the reference CBS interaction energy value. They interpreted the outcome such that augmentation functions likely improve the tails of trial wave functions that are crucial for describing the weak interactions correctly.^34^ The wider set of results reported in this work supports the above interpretation. The interaction among molecules included in the A24 data set are categorized into three groups:^48^ Hydrogen bonds (index 1 to 5), mixed interactions (index 6 to 15), and dispersion-dominated interactions (index 16 to 24). Dimers in the hydrogen-bond group show the most significant BSIEs, while the dispersion-dominated dimers are less affected by BSIEs. The hydrogen bond, which originates from the Coulomb interactions, has the long-tail effect (e.g., 1/*r*) compared with the dispersion-dominated ones, which are typically shorter-range interactions (e.g., 1/*r*^6^). It appears that the long-tail of the interaction has an effect on the nodal surface (affecting the FN-DMC evaluations), which can be improved if diffuse functions are available in the basis set.

In Figure 2, the comparison between BSIEs with cc-pVDZ with and without the CP correction of the basis set shows that this correction alleviates the BSIEs. It implies that the basis sets assigned to the ghost atoms can compensate missing diffuse functions in the cc-pVDZ basis set, thus improving the nodal surface of the monomers and decreasing the FN error on the binding energy evaluations. This suggests that the CP correction is an alternative way to reduce BSIEs in DMC calculations. The simultaneous use of augmentation and CP leads to a synergistic effect, as can be appreciated in Figure 2 observing the evaluations obtained using aug-cc-pVDZ with CP.

Figure 3(a) summarizes the BSIEs obtained with all the family members of the cc basis sets and PW used in this study. The left panel of Figure 3(a) plots the BSIEs with the nonaugmented cc basis sets (cc-pV*n*Z: *n* = D,T,Q,5,6), revealing that, to get binding energies in the CBS limit within their statistical errors (3 σ ∼ 0.25 kcal/mol), one needs the cc-pVQZ without the CP corrections or the cc-pVTZ with the CP correction. The cc-pVTZ without the CP correction introduces non-negligible BSIEs in binding energies of several molecules in the A24 set (see. Table SIII of the SI), such as water–ammonia, ammonia dimer, and formaldehyde–ethene. The central panel of Figure 3(a) plots BSIEs with the augmented cc basis sets (aug-cc-pV*n*Z: *n* = D,T,Q,5), indicating that the augmentations of the basis sets improve the situation. To get binding energies in the CBS limit within their statistical errors, one needs the aug-cc-pVTZ without the CP correction or the aug-cc-pVDZ basis with the CP correction. The right panel of Figure 3(a) plots BSIEs with PW basis set, confirming that aug-cc-pV6Z basis set gives binding energies in the CBS limit (i.e., zero BSIEs within the statistical errors).

It is informative to compare the BSIEs obtained by DMC with those obtained using a quantum chemistry method, such as MP2, to understand the impact of basis sets. The comparison between Figure 3(a),(b) reveals that BSIEs in the DMC calculations are not as significant as in the MP2 calculations, as believed in the QMC community. This is true not only for the binding energies, but also the total energies of fragments and complexes, as shown in Figure S3. In panel(b), the asymptotic behaviors with *n* are seen in the binding energies computed by MP2. For QC calculations, the most common and established procedure to reach the CBS limit is the extrapolation of the binding energies with consecutive cardinal numbers.^65^ The asymptotic behaviors allow the extrapolation, and, in fact, the CP correction in MP2 calculations is necessary for smoother extrapolations to the CBS limit (c.f. Figures S8 and S9 of the SI plot *E*_b_ for all 24 systems), as already mentioned in ref (44). Instead, in DMC calculations, the extrapolation is no longer needed, as shown in panel (a) (c.f. Figures S10 and S11 plot *E*_b_ for all 24 systems). The observed differences between MP2 and DMC likely arise because the energy from the latter is affected only by the nodal surface, while the former depends on the entire wave function. While BSIEs are correlated among these methods, the correlation is inadequate for discussions at the subchemical accuracy level (see Figure S16 of the SI). In other words, we found that the BSIEs in DMC calculations cannot be estimated accurately from those in other QC calculations.

Our study reveals that, in DMC calculations, one can get the binding energy in the CBS limit only with a single medium-size basis set (such as cc-pVQZ and aug-cc-pVTZ). It helps decrease a DMC computational cost to reach the CBS limit because we can avoid using atomic orbitals with higher angular momenta (e.g., *h* and *i* orbitals).^3^ For instance, the LRDMC computational cost of the water dimer with respect to basis set families is plotted in Figure S17. It indicates that the aug-cc-pVTZ basis set strikes a good balance between the computational cost and BSIE in the binding energy calculation. Furthermore, it should be emphasized that the use of a medium-size basis set is also important from the perspective of reducing memory requirements because memory limitation can be critical for large systems rather than the computational cost.

In summary, we revealed that both BSSE and BSIE are not negligible in DMC binding energy calculations if one targets to compute binding energies of complex systems within the subchemical accuracy (i.e., ∼0.1 kcal/mol). The augmentation (i.e., more diffuse functions) of a basis set and the CP correction for a basis set are both helpful to reduce BSIEs, i.e., to get binding energies in the CBS limit.

## 5 A24 Benchmark Test Revisited

Benchmarks for the A24 set were done by Dubecký et al.^35^ and by Nakano et al.^67^ with the aug-TZV basis sets associated with the ECPs developed by Burkatzki et al.^68^ and the cc-pVTZ basis sets associated with the ECPs developed by Bennett et al.,^49,50^ respectively. Root mean square deviation (RMSD) of the binding energies from CCSD(T) reported by Dubecký et al.^35^ and Nakano et al.^67^ are 0.15 and 0.315 kcal/mol, respectively. Table 1 shows the binding energies obtained in this study by DMC calculations with the aug-ccpV6Z basis sets (without CP) associated with the ECPs developed by Bennett et al.,^49,50^ and those obtained by CCSD(T) in the CBS limit taken from Benchmark Energy and Geometry DataBase (BEGDB).^69^ In this work, we obtained a RMSD of 0.135 kcal/mol, which is very close to the value obtained by Dubecký, while ∼0.2 kcal/mol off from the value reported by Nakano et al. As mentioned in the previous section, Figure 3(a) indicates that the cc-pVTZ basis set without the CP correction shows non-negligible BSIEs and the augmentation (aug-ccpVTZ) reduces the BSIEs significantly. In fact, we got 0.247(14) and 0.131(14) kcal/mol for RMSD with cc-pVTZ and aug-ccpVTZ basis sets, respectively. The obtained cc-pVTZ value (0.247(14) kcal/mol) is very close to those previously reported by Nakano et al.^67^ (0.315 kcal/mol), although the treatments of the nonlocal terms are different (DLA was employed in the previous study, while DTM is employed in the present study). As such, the RMSD obtained by Nakano et al.^67^ should be a little affected by BSIEs, while the values obtained by Dubecký et al.^35^ with the augmented basis sets should already reach the CBS limit. Thus, as the benchmark values for the A24 data set, one should refer to the binding energies obtained by Dubecký et al.^35^ or those obtained in this work.

## 6 Conclusions

In this study, we investigated two basis-set related errors, BSIEs and BSSEs, in binding energy calculations by ab initio FN-DMC calculations using the A24 benchmark set. We revealed that BSIE and BSSE are not negligible in DMC calculations when a small basis set, such as cc-pVDZ and cc-pVTZ, is used without the CP correction. Our study implies that, to get binding energies in the CBS limit with GTOs, one should use, at least, a medium-size basis set, such as cc-pVQZ or aug-cc-pVTZ basis set. We found that the CP correction is also helpful in DMC calculations to reduce BSIEs, as in QC calculations. With the CP correction, one can use a smaller basis, such as cc-pVTZ or aug-cc-pVDZ basis sets. This work raises awareness of BSSEs and BSIEs in binding energy calculations by DMC, which have not been extensively studied previously. In the future, it would be interesting to perform a more comprehensive study investigating BSIEs in DMC for larger molecules or periodic systems.

## Data Availability

The QMC kernels used in this work, TurboRVB and QMCPACK, are available from their GitHub repositories, [https://github.com/sissaschool/turborvb] and [https://github.com/QMCPACK/qmcpack], respectively.

## Supporting Information Available

Figures plotting binding energies obtained by the methods employed in this study (HF, MP2, and DMC) with various basis sets (PDF)

## Footnotes

1 Note that PW basis sets are not affected by any BSSE, while they can be affected by a BSIE when the PW cutoff is too small.

2 Note that *E*_b_^CP^ ≥ *E*_b_, because *E*_b_^BSSE^ ≤ 0 as *M*^AB^ > *M*^A^ and *M*^AB^ > *M*^B^.

3 A DMC computation with the spline basis is independent of a chosen grid-size,^66^ while its memory requirement scales.

## References

[ref1] Ceperley D. M. The statistical error of green’s function Monte Carlo. J. Stat. Phys. 1986, 43, 815–826. 10.1007/BF02628307.

[ref2] Foulkes W. M. C.; Mitas L.; Needs R. J.; Rajagopal G. Quantum Monte Carlo simulations of solids. Rev. Mod. Phys. 2001, 73, 33 10.1103/RevModPhys.73.33.

[ref3] Drummond N. D.; Monserrat B.; Lloyd-Williams J. H.; Ríos P. L.; Pickard C. J.; Needs R. J. Quantum Monte Carlo study of the phase diagram of solid molecular hydrogen at extreme pressures. Nat. Commun. 2015, 6, 7794 10.1038/ncomms8794. 26215251 PMC4525154

[ref4] Mazzola G.; Helled R.; Sorella S. Phase diagram of hydrogen and a hydrogen-helium mixture at planetary conditions by Quantum Monte Carlo simulations. Phys. Rev. Lett. 2018, 120, 025701 10.1103/PhysRevLett.120.025701. 29376719

[ref5] Tirelli A.; Tenti G.; Nakano K.; Sorella S. High-pressure hydrogen by machine learning and quantum Monte Carlo. Phys. Rev. B 2022, 106, L041105 10.1103/PhysRevB.106.L041105.

[ref6] Ly K. K.; Ceperley D. M. Phonons of metallic hydrogen with quantum Monte Carlo. J. Chem. Phys. 2022, 156, 044108 10.1063/5.0077749. 35105096

[ref7] Niu H.; Yang Y.; Jensen S.; Holzmann M.; Pierleoni C.; Ceperley D. M. Stable Solid Molecular Hydrogen above 900 K from a Machine-Learned Potential Trained with Diffusion Quantum Monte Carlo. Phys. Rev. Lett. 2023, 130, 076102 10.1103/PhysRevLett.130.076102. 36867819

[ref8] Monacelli L.; Casula M.; Nakano K.; Sorella S.; Mauri F. Quantum phase diagram of high-pressure hydrogen. Nat. Phys. 2023, 19, 845–850. 10.1038/s41567-023-01960-5.

[ref9] Tenti G.; Nakano K.; Tirelli A.; Sorella S.; Casula M. Principal deuterium Hugoniot via quantum Monte Carlo and Δ−learning. Phys. Rev. B 2024, 110, L041107 10.1103/PhysRevB.110.L041107.

[ref10] Krogel J. T.; Yuk S. F.; Kent P. R. C.; Cooper V. R. Perspectives on van der Waals Density Functionals: The Case of TiS_2_. J. Phys. Chem. A 2020, 124, 9867–9876. 10.1021/acs.jpca.0c05973. 33190498

[ref11] Ichibha T.; Dzubak A. L.; Krogel J. T.; Cooper V. R.; Reboredo F. A. CrI_3_ revisited with a many-body ab initio theoretical approach. Phys. Rev. Mater. 2021, 5, 064006 10.1103/PhysRevMaterials.5.064006.

[ref12] Nikaido Y.; Ichibha T.; Hongo K.; Reboredo F. A.; Kumar K. C. H.; Mahadevan P.; Maezono R.; Nakano K. Diffusion Monte Carlo Study on Relative Stabilities of Boron Nitride Polymorphs. J. Phys. Chem. C 2022, 126, 6000–6007. 10.1021/acs.jpcc.1c10943.

[ref13] Wines D.; Choudhary K.; Tavazza F. Systematic DFT+U and Quantum Monte Carlo Benchmark of Magnetic Two-Dimensional (2D) CrX_3_ (X = I, Br, Cl, F). J. Phys. Chem. C 2023, 127, 1176–1188. 10.1021/acs.jpcc.2c06733. PMC9888057 36727030

[ref14] Wines D.; Tiihonen J.; Saritas K.; Krogel J. T.; Ataca C. A Quantum Monte Carlo Study of the Structural, Energetic, and Magnetic Properties of Two-Dimensional H and T Phase VSe_2_. J. Phys. Chem. Lett. 2023, 14, 3553–3560. 10.1021/acs.jpclett.3c00497. 37017431

[ref15] Zen A.; Brandenburg J. G.; Klimeš J.; Tkatchenko A.; Alfè D.; Michaelides A. Fast and accurate quantum Monte Carlo for molecular crystals. Proc. Natl. Acad. Sci. U.S.A. 2018, 115, 1724–1729. 10.1073/pnas.1715434115. 29432177 PMC5828600

[ref16] Della Pia F.; Zen A.; Alfè D.; Michaelides A. How Accurate Are Simulations and Experiments for the Lattice Energies of Molecular Crystals?. Phys. Rev. Lett. 2024, 133, 046401 10.1103/PhysRevLett.133.046401. 39121404

[ref17] Beaudet T. D.; Casula M.; Kim J.; Sorella S.; Martin R. M. Molecular hydrogen adsorbed on benzene: Insights from a quantum Monte Carlo study. J. Chem. Phys. 2008, 129, 164711 10.1063/1.2987716. 19045302

[ref18] Zen A.; Roch L. M.; Cox S. J.; Hu X. L.; Sorella S.; Alfè D.; Michaelides A. Toward accurate adsorption energetics on clay surfaces. J. Phys. Chem. C 2016, 120, 26402–26413. 10.1021/acs.jpcc.6b09559. PMC5126707 27917256

[ref19] Al-Hamdani Y. S.; Rossi M.; Alfè D.; Tsatsoulis T.; Ramberger B.; Brandenburg J. G.; Zen A.; Kresse G.; Grüneis A.; Tkatchenko A.; Michaelides A. Properties of the water to boron nitride interaction: From zero to two dimensions with benchmark accuracy. J. Chem. Phys. 2017, 147, 044710 10.1063/1.4985878. 28764374

[ref20] Hsing C.-R.; Chang C.-M.; Cheng C.; Wei C.-M. Quantum Monte Carlo Studies of CO Adsorption on Transition Metal Surfaces. J. Phys. Chem. C 2019, 123, 15659–15664. 10.1021/acs.jpcc.9b03780.

[ref21] Shi B. X.; Zen A.; Kapil V.; Nagy P. R.; Grüneis A.; Michaelides A. Many-Body Methods for Surface Chemistry Come of Age: Achieving Consensus with Experiments. J. Am. Chem. Soc. 2023, 145, 25372–25381. 10.1021/jacs.3c09616. 37948071 PMC10683001

[ref22] Bressanini D.; Morosi G. On the nodal structure of single-particle approximation based atomic wave functions. J. Chem. Phys. 2008, 129, 054103 10.1063/1.2963501. 18698884

[ref23] Morales M. A.; McMinis J.; Clark B. K.; Kim J.; Scuseria G. E. Multideterminant wave functions in quantum Monte Carlo. J. Chem. Theory Comput. 2012, 8, 2181–2188. 10.1021/ct3003404. 26588949

[ref24] Nakano K.; Sorella S.; Alfè D.; Zen A. Beyond Single-Reference Fixed-Node Approximation in Ab Initio Diffusion Monte Carlo Using Antisymmetrized Geminal Power Applied to Systems with Hundreds of Electrons. J. Chem. Theory Comput. 2024, 20, 4591–4604. 10.1021/acs.jctc.4c00139. 38788330 PMC11171267

[ref25] Huang B.; von Lilienfeld O. A.; Krogel J. T.; Benali A. Toward DMC accuracy across chemical space with scalable Δ−QML. J. Chem. Theory Comput. 2023, 19, 1711–1721. 10.1021/acs.jctc.2c01058. 36857531

[ref26] Korth M.; Grimme S.; Towler M. D. The Lithium-Thiophene Riddle Revisited. J. Phys. Chem. A 2011, 115, 11734–11739. 10.1021/jp204132g. 21877699

[ref27] Nemec N.; Towler M. D.; Needs R. J. Benchmark all-electron ab initio quantum Monte Carlo calculations for small molecules. J. Chem. Phys. 2010, 132, 034111 10.1063/1.3288054. 20095732

[ref28] Petruzielo F. R.; Toulouse J.; Umrigar C. J. Approaching chemical accuracy with quantum Monte Carlo. J. Chem. Phys. 2012, 136, 124116 10.1063/1.3697846. 22462844

[ref29] Zen A.; Luo Y.; Sorella S.; Guidoni L. Molecular Properties by Quantum Monte Carlo: An Investigation on the Role of the Wave Function Ansatz and the Basis Set in the Water Molecule. J. Chem. Theory Comput. 2013, 9, 4332–4350. 10.1021/ct400382m. 24526929 PMC3920371

[ref30] Scemama A.; Applencourt T.; Giner E.; Caffarel M. Quantum Monte Carlo with very large multideterminant wavefunctions. J. Comput. Chem. 2016, 37, 1866–1875. 10.1002/jcc.24382. 27302337

[ref31] Caffarel M.; Applencourt T.; Giner E.; Scemama A. Communication: Toward an improved control of the fixed-node error in quantum Monte Carlo: The case of the water molecule. J. Chem. Phys. 2016, 144, 151103 10.1063/1.4947093. 27389201

[ref32] Scemama A.; Giner E.; Benali A.; Loos P.-F. Taming the fixed-node error in diffusion Monte Carlo via range separation. J. Chem. Phys. 2020, 153, 174107 10.1063/5.0026324. 33167659

[ref33] Korth M.; Lüchow A.; Grimme S. Toward the exact solution of the electronic Schrödinger equation for noncovalent molecular interactions: worldwide distributed quantum Monte Carlo calculations. J. Phys. Chem. A 2008, 112, 2104–2109. 10.1021/jp077592t. 18201073

[ref34] Dubecký M.; Jurecka P.; Derian R.; Hobza P.; Otyepka M.; Mitas L. Quantum Monte Carlo methods describe noncovalent interactions with subchemical accuracy. J. Chem. Theory Comput. 2013, 9, 4287–4292. 10.1021/ct4006739. 26589147

[ref35] Dubecký M.; Derian R.; Jurečka P.; Mitas L.; Hobza P.; Otyepka M. Quantum Monte Carlo for noncovalent interactions: an efficient protocol attaining benchmark accuracy. Phys. Chem. Chem. Phys. 2014, 16, 20915–20923. 10.1039/C4CP02093F. 25170978

[ref36] Mostaani E.; Drummond N. D.; Fal’ko V. I. Quantum Monte Carlo Calculation of the Binding Energy of Bilayer Graphene. Phys. Rev. Lett. 2015, 115, 115501 10.1103/PhysRevLett.115.115501. 26406840

[ref37] Nakano K.; Maezono R.; Sorella S. All-Electron Quantum Monte Carlo with Jastrow Single Determinant Ansatz: Application to the Sodium Dimer. J. Chem. Theory Comput. 2019, 15, 4044–4055. 10.1021/acs.jctc.9b00295. 31117480

[ref38] Benali A.; Shin H.; Heinonen O. Quantum Monte Carlo benchmarking of large noncovalent complexes in the L7 benchmark set. J. Chem. Phys. 2020, 153, 194113 10.1063/5.0026275. 33218249

[ref39] Al-Hamdani Y. S.; Nagy P. R.; Zen A.; Barton D.; Kállay M.; Brandenburg J. G.; Tkatchenko A. Interactions between large molecules pose a puzzle for reference quantum mechanical methods. Nat. Commun. 2021, 12, 3927 10.1038/s41467-021-24119-3. 34168142 PMC8225865

[ref40] Raghav A.; Maezono R.; Hongo K.; Sorella S.; Nakano K. Toward Chemical Accuracy Using the Jastrow Correlated Antisymmetrized Geminal Power Ansatz. J. Chem. Theory Comput. 2023, 19, 2222–2229. 10.1021/acs.jctc.2c01141. 37014742 PMC10134432

[ref41] Dubecký M.; Mitas L.; Jurečka P. Noncovalent Interactions by Quantum Monte Carlo. Chem. Rev. 2016, 116, 5188–5215. 10.1021/acs.chemrev.5b00577. 27081724

[ref42] Schäfer T.; Irmler A.; Gallo A.; Grüneis A. Understanding Discrepancies of Wavefunction Theories for Large Molecules. 2024, arXiv:2407.01442v3. arXiv.org e-Print archive https://doi.org/10.48550/arXiv.2407.01442. 10.1038/s41467-025-64104-8 PMC12521354 41087329

[ref43] Van Duijneveldt F. B.; van Duijneveldt-van de Rijdt J. G.; van Lenthe J. H. State of the art in counterpoise theory. Chem. Rev. 1994, 94, 1873–1885. 10.1021/cr00031a007.

[ref44] Dunning T. H. A road map for the calculation of molecular binding energies. J. Phys. Chem. A 2000, 104, 9062–9080. 10.1021/jp001507z.

[ref45] Dunning T. H. Jr Gaussian basis sets for use in correlated molecular calculations. I. The atoms boron through neon and hydrogen. J. Chem. Phys. 1989, 90, 1007–1023. 10.1063/1.456153.

[ref46] Boys S.; Bernardi F. The calculation of small molecular interactions by the differences of separate total energies. Some procedures with reduced errors. Mol. Phys. 1970, 19, 553–566. 10.1080/00268977000101561.

[ref47] Zhou X.; Huang Z.; He X. Diffusion Monte Carlo method for barrier heights of multiple proton exchanges and complexation energies in small water, ammonia, and hydrogen fluoride clusters. J. Chem. Phys. 2024, 160, 054103 10.1063/5.0182164. 38310472

[ref48] Řezáč J.; Hobza P. Describing noncovalent interactions beyond the common approximations: how accurate is the “gold standard,” CCSD (T) at the complete basis set limit?. J. Chem. Theory Comput. 2013, 9, 2151–2155. 10.1021/ct400057w. 26583708

[ref49] Bennett M. C.; Melton C. A.; Annaberdiyev A.; Wang G.; Shulenburger L.; Mitas L. A new generation of effective core potentials for correlated calculations. J. Chem. Phys. 2017, 147, 224106 10.1063/1.4995643. 29246065

[ref50] Bennett M. C.; Wang G.; Annaberdiyev A.; Melton C. A.; Shulenburger L.; Mitas L. A new generation of effective core potentials from correlated calculations: 2nd row elements. J. Chem. Phys. 2018, 149, 104108 10.1063/1.5038135. 30219005

[ref51] Nakano K.; Attaccalite C.; Barborini M.; Capriotti L.; Casula M.; Coccia E.; Dagrada M.; Genovese C.; Luo Y.; Mazzola G.; Zen A.; Sorella S. TurboRVB: A many-body toolkit for ab initio electronic simulations by quantum Monte Carlo. J. Chem. Phys. 2020, 152, 204121 10.1063/5.0005037. 32486669

[ref52] Casula M.; Filippi C.; Sorella S. Diffusion Monte Carlo method with lattice regularization. Phys. Rev. Lett. 2005, 95, 100201 10.1103/PhysRevLett.95.100201. 16196912

[ref53] Zen A.; Brandenburg J. G.; Michaelides A.; Alfè D. A new scheme for fixed node diffusion quantum Monte Carlo with pseudopotentials: Improving reproducibility and reducing the trial-wave-function bias. J. Chem. Phys. 2019, 151, 134105 10.1063/1.5119729. 31594339

[ref54] Posenitskiy E.; Chilkuri V. G.; Ammar A.; Hapka M.; Pernal K.; Shinde R.; Borda E. J. L.; Filippi C.; Nakano K.; Kohulák O.; Sorella S.; de Oliveira Castro P.; Jalby W.; Ríos P. L.; Alavi A.; Scemama A. TREXIO: A file format and library for quantum chemistry. J. Chem. Phys. 2023, 158, 174801 10.1063/5.0148161. 37144717

[ref55] Sun Q.; Berkelbach T. C.; Blunt N. S.; Booth G. H.; Guo S.; Li Z.; Liu J.; McClain J. D.; Sayfutyarova E. R.; Sharma S.; et al. PySCF: the Python-based simulations of chemistry framework. WIREs Comput. Mol. Sci. 2018, 8, e1340 10.1002/wcms.1340.

[ref56] Sun Q.; Zhang X.; Banerjee S.; Bao P.; Barbry M.; Blunt N. S.; Bogdanov N. A.; Booth G. H.; Chen J.; Cui Z. H.; Eriksen J. J.; Gao Y.; Guo S.; Hermann J.; Hermes M. R.; Koh K.; Koval P.; Lehtola S.; Li Z.; Liu J.; Mardirossian N.; McClain J. D.; Motta M.; Mussard B.; Pham H. Q.; Pulkin A.; Purwanto W.; Robinson P. J.; Ronca E.; Sayfutyarova E. R.; Scheurer M.; Schurkus H. F.; Smith J. E.; Sun C.; Sun S. N.; Upadhyay S.; Wagner L. K.; Wang X.; White A.; Whitfield J. D.; Williamson M. J.; Wouters S.; Yang J.; Yu J. M.; Zhu T.; Berkelbach T. C.; Sharma S.; Sokolov A. Y.; Chan G. K. L. Recent developments in the PySCF program package. J. Chem. Phys. 2020, 153, 024109 10.1063/5.0006074. 32668948

[ref57] Perdew J. P.; Zunger A. Self-interaction correction to density-functional approximations for many-electron systems. Phys. Rev. B 1981, 23, 5048 10.1103/PhysRevB.23.5048.

[ref58] Sorella S. Green function Monte Carlo with stochastic reconfiguration. Phys. Rev. Lett. 1998, 80, 4558 10.1103/PhysRevLett.80.4558.

[ref59] Kim J.; Baczewski A. D.; Beaudet T. D.; Benali A.; Bennett M. C.; Berrill M. A.; Blunt N. S.; Borda E. J. L.; Casula M.; Ceperley D. M.; Chiesa S.; Clark B. K.; Clay R. C.; Delaney K. T.; Dewing M.; Esler K. P.; Hao H.; Heinonen O.; Kent P. R. C.; Krogel J. T.; Kylänpää I.; Li Y. W.; Lopez M. G.; Luo Y.; Malone F. D.; Martin R. M.; Mathuriya A.; McMinis J.; Melton C. A.; Mitas L.; Morales M. A.; Neuscamman E.; Parker W. D.; Flores S. D. P.; Romero N. A.; Rubenstein B. M.; Shea J. A. R.; Shin H.; Shulenburger L.; Tillack A. F.; Townsend J. P.; Tubman N. M.; Goetz B. V. D.; Vincent J. E.; Yang D. C.; Yang Y.; Zhang S.; Zhao L. QMCPACK: an open sourceab initioquantum Monte Carlo package for the electronic structure of atoms, molecules and solids. J. Phys.: Condens. Matter 2018, 30, 195901 10.1088/1361-648X/aab9c3. 29582782

[ref60] Kent P. R. C.; Annaberdiyev A.; Benali A.; Bennett M. C.; Borda E. J. L.; Doak P.; Hao H.; Jordan K. D.; Krogel J. T.; Kylänpaä I.; Lee J.; Luo Y.; Malone F. D.; Melton C. A.; Mitas L.; Morales M. A.; Neuscamman E.; Reboredo F. A.; Rubenstein B.; Saritas K.; Upadhyay S.; Wang G.; Zhang S.; Zhao L. QMCPACK: Advances in the development, efficiency, and application of auxiliary field and real-space variational and diffusion quantum Monte Carlo. J. Chem. Phys. 2020, 152, 174105 10.1063/5.0004860. 32384844

[ref61] Perdew J. P.; Burke K.; Ernzerhof M. Generalized Gradient Approximation Made Simple. Phys. Rev. Lett. 1996, 77, 3865 10.1103/PhysRevLett.77.3865. 10062328

[ref62] Adamo C.; Barone V. Toward Reliable Density Functional Methods without Adjustable Parameters: The PBE0Model. J. Chem. Phys. 1999, 110, 6158–6170. 10.1063/1.478522.

[ref63] Becke A. D. Density-functional Thermochemistry. III. The Role of Exact Exchange. J. Chem. Phys. 1993, 98, 5648–5652. 10.1063/1.464913.

[ref64] Mardirossian N.; Head-Gordon M. *ω*B97M-V: A Combinatorially Optimized, Range-Separated Hybrid, Meta-GGA Density Functional with VV10 Nonlocal Correlation. J. Chem. Phys. 2016, 144, 214110 10.1063/1.4952647. 27276948

[ref65] Neese F.; Valeev E. F. Revisiting the Atomic Natural Orbital Approach for Basis Sets: Robust Systematic Basis Sets for Explicitly Correlated and Conventional Correlated Ab Initio Methods?. J. Chem. Theory Comput. 2011, 7, 33–43. 10.1021/ct100396y. 26606216

[ref66] Alfè D.; Gillan M. J. Efficient localized basis set for quantum Monte Carlo calculations on condensed matter. Phys. Rev. B 2004, 70, 161101 10.1103/PhysRevB.70.161101.

[ref67] Nakano K.; Kohulák O.; Raghav A.; Casula M.; Sorella S. TurboGenius: Python suite for high-throughput calculations of ab initio quantum Monte Carlo methods. J. Chem. Phys. 2023, 159, 224801 10.1063/5.0179003. 38078530

[ref68] Burkatzki M.; Filippi C.; Dolg M. Energy-consistent pseudopotentials for quantum Monte Carlo calculations. J. Chem. Phys. 2007, 126, 234105 10.1063/1.2741534. 17600402

[ref69] Řezáč J.; Jurečka P.; Riley K. E.; Černỳ J.; Valdes H.; Pluháčková K.; Berka K.; Řezáč T.; Pitoňák M.; Vondrášek J.; Hobza P. Quantum chemical benchmark energy and geometry database for molecular clusters and complex molecular systems (www. begdb. com): a users manual and examples. Collect. Czech. Chem. Commun. 2008, 73, 1261–1270. 10.1135/cccc20081261.

