# Supplementary material for: Basis Set Incompleteness Errors in Fixed-Node Diffusion Monte Carlo Calculations on Noncovalent Interactions
Source: J Chem Theory Comput. 2025 Apr 30;21(9):4426–34. doi: 10.1021/acs.jctc.4c01631 (PMC12079793; doi:10.1021/acs.jctc.4c01631)
Supplement: Supplementary file 1 — ct4c01631_si_001.pdf [file ct4c01631_si_001.pdf]

Supplementary Material for Basis set incompleteness errors in fixed-node diffusion Monte Carlo calculations on non-covalent interactions

Kousuke Nakano,*,† Benjamin X. Shi,‡ Dario Alfè,¶,§,‖ and Andrea Zen*,¶,§

†*Center for Basic Research on Materials, National Institute for Materials Science (NIMS), Tsukuba, Ibaraki 305-0047, Japan*

‡*Yusuf Hamied Department of Chemistry, University of Cambridge, Cambridge CB2 1EW, United Kingdom*

¶*Dipartimento di Fisica Ettore Pancini, Università di Napoli Federico II, Monte S. Angelo, I-80126 Napoli, Italy*

§*Department of Earth Sciences, University College London, Gower Street, London WC1E 6BT, United Kingdom*

‖*Thomas Young Centre and London Centre for Nanotechnology, 17-19 Gordon Street, London WC1H 0AH, United Kingdom*

E-mail: kousuke_1123@icloud.com; andrea.zen@unina.it

Abstract

This file includes the supplementary information for the paper titled "Basis set incompleteness errors in fixed-node diffusion Monte Carlo calculations on non-covalent interactions".

List of Figures

List of Tables

SI The binding energies (in kcal/mol) of the molecule contained in the A24-set, as obtained from FN-DMC evaluations using QMCPACK and the setup described in section 1. . . . . . s7

SII The total energies (E^{tot}) and variances (σ^2) of the wavefunctions for the water–ammonia dimer computed using VMC. s9

SIII The binding energies (in kcal/mol) of the molecule contained in the A24-set, as obtained from FN-DMC evaluations using TurboRVB with the ccecp-cc-pVnZ (VnZ) basis set family (n=D, T, Q, 5, 6) without CP correction. s10

SIV The binding energies (in kcal/mol) of the molecule contained in the A24-set, as obtained from FN-DMC evaluations using TurboRVB with the ccecp-aug-cc-pVnZ (aVnZ) basis set family (n=D, T, Q, 5, 6) without CP correction. s11

SV The binding energies (in kcal/mol) of ammonia dimer with LDA-PZ and B3LYP nodal surfaces, as obtained from FN-DMC evaluations using TurboRVB with the ccecp-cc-pVnZ (VnZ; n=D, T) and ccecp-aug-cc-pVnZ (aVnZ; n=D, T, Q, 5, 6) basis set families using the setup described in the main tex. $\Delta = E_{\mathrm{b}}^{\mathrm{LDA-PZ}} - E_{\mathrm{b}}^{\mathrm{B3LYP}}$. s11

SVI The binding energies (in kcal/mol) of ammonia dimer with LDA-PZ, PBE, ωB97M-V, PBE0, B3LYP and HF nodal surfaces, as obtained from FN-DMC evaluations using QMCPACK with the ccecp-cc-pVnZ (VnZ; n=D, T, Q, 5, 6) and ccecp-aug-cc-pVnZ (aVnZ; n=D, T, Q, 5, 6) basis set families using the setup described in Sec. 1 for a $\tau = 0.003\,\mathrm{au}$ timestep. s12

1 Additional QMCPACK calculations

QMCPACK[1,2] is a high-performance real space QMC code capable of performing molecular and solid state calculations on modern CPU and GPU machines. QMCPACK implements the standard (time-discretized) FN-DMC algorithm, as well as the variational QMC algorithm and several schemes for the trial wavefunction optimization.

QMCPACK simulations were performed using a Slater-Jastrow trial wavefunction, with the determinant part of the wavefunction being a single Slater determinant, and employing the ccECP pseudopotentials[3,4] (as for to the TurboRVB simulations). The Slater determinant is obtained from a preliminary DFT calculation employing the PZ-LDA[5] exchange-correlation functional. We performed two sets of simulations, using localized GTO basis sets or PW basis sets to express the DFT single-particle orbitals. Five sets of calculations were done for the GTO trial wavefunctions, using the following basis sets (designed for the ccECP pseudopotentials): ccecp-cc-pVDZ (VDZ), ccecp-aug-cc-pVDZ (aVDZ), ccecp-cc-pVTZ (VTZ), ccecp-aug-cc-pVTZ (aVTZ), and ccecp-aug-cc-pV6Z (aV6Z). The preliminary DFT calculation was performed using the PySCF[6,7] package. To generate plane-wave (PW) based trial wavefunctions we used the Quantum ESPRESSO[8] package with a 600 Ryd energy cutoff. The single-particle orbitals obtained are then reexpanded in terms of B-splines[9] in the QMC calculations.

We used the default Jastrow factor implemented in QMCPACK, which includes electron-nucleus, electron-electron, and electron-electron-nucleus terms. The electron-nucleus and electron-electron functions are both one-dimensional B-spline (tricubic spline on a linear grid) between zero and a cutoff distance. The electron-electron-nucleus function is a polynomial expansion. The parameters of the Jastrow factor have been optimised by minimising the variational energy of each system, employing the linear method with line minimization via quartic polynomial fits, and performing several steps of optimization with a sampling of up to 10,000,000 configurations.

The DMC calculations were performed with the T-move localization scheme.[10] DMC

simulations have been performed with a target population of 102,400 walkers, and employing the modification to the drift and branching terms suggested in Ref. 11, called with the flag `ZSGMA`. The reason for using the T-move localization scheme in QMCPACK calculations, instead of the DTM scheme used in the TurboRVB evaluations is that the available version (version 3.17.1, released on 25 August 2023) and previous versions of QMCPACK are affected by a bug on the DTM implementation. Note that the different scheme used for the non-local psudopotential terms in QMCPACK and TurboRVB calculations implies that the QMCPACK-based evaluations (i.e., with T-move) and TurboRVB-based evaluations (i.e., with DTM) are not equivalent. The reported DMC results are obtained for a timestep $\tau = 0.01$ au. Some additional DMC simulations with $\tau = 0.03$ au and $\tau = 0.003$ au show that the $\tau = 0.01$ au evaluations of the binding energy have a negligible timestep bias (namely, the timestep bias is smaller than the stochastic error).

DMC evaluations of the binding energies obtained using the above setup are reported in Table SI. The BSIE of the evaluations are shown in Figure S1.

Table SI: The binding energies (in kcal/mol) of the molecule contained in the A24-set, as obtained from FN-DMC evaluations using QMCPACK and the setup described in section 1.

	VDZ	VTZ	aVDZ	aVTZ	aV6Z	PW
water–ammonia	-7.74(6)	-7.15(6)	-6.85(6)	-6.69(5)	-6.61(7)	-6.69(6)
water dimer	-5.74(7)	-5.37(6)	-5.39(5)	-5.27(5)	-5.15(5)	-5.24(6)
HCN dimer	-5.48(7)	-5.39(10)	-5.35(8)	-5.12(7)	-5.03(8)	-5.04(7)
HF dimer	-5.08(6)	-4.86(6)	-4.72(5)	-4.78(5)	-4.81(5)	-4.82(5)
ammonia dimer	-4.30(9)	-3.56(6)	-3.11(7)	-3.16(5)	-3.17(5)	-3.30(7)
HF–methane	-1.62(5)	-1.82(9)	-1.66(5)	-1.62(5)	-1.70(6)	-1.58(5)
ammonia–methane	-1.40(6)	-1.11(5)	-0.89(5)	-0.83(6)	-0.76(6)	-0.80(5)
water–methane	-1.02(6)	-0.75(6)	-0.67(5)	-0.67(5)	-0.69(5)	-0.67(5)
formaldehyde dimer	-4.89(8)	-4.80(8)	-4.69(10)	-4.64(8)	-4.58(9)	-4.59(14)
water–ethene	-2.55(8)	-2.80(8)	-2.83(7)	-2.65(8)	-2.54(6)	-2.62(8)
formaldehyde–ethene	-2.03(8)	-1.88(8)	-1.99(8)	-1.46(8)	-1.60(8)	-1.56(7)
ethyne dimer	-1.86(9)	-1.54(7)	-1.63(7)	-1.51(7)	-1.56(7)	-1.61(8)
ammonia–ethene	-1.62(8)	-1.59(7)	-1.58(8)	-1.42(8)	-1.46(8)	-1.46(6)
ethene dimer	-1.25(9)	-1.07(8)	-0.97(9)	-0.98(6)	-0.89(7)	-1.13(8)
methane–ethene	-0.70(6)	-0.48(7)	-0.71(7)	-0.58(6)	-0.44(10)	-0.57(6)
borane–methane	-1.22(5)	-1.41(5)	-1.31(7)	-1.34(7)	-1.51(5)	-1.39(5)
methane–ethane	-0.92(7)	-0.95(8)	-0.93(5)	-0.83(9)	-0.70(7)	-0.72(9)
methane–ethane	-0.83(8)	-0.57(9)	-0.76(7)	-0.63(6)	-0.63(6)	-0.57(10)
methane dimer	-0.64(7)	-0.55(4)	-0.63(6)	-0.51(5)	-0.62(5)	-0.54(5)
Ar–methane	-0.17(4)	-0.07(4)	-0.21(4)	-0.43(4)	-0.15(5)	-0.01(8)
Ar–ethene	0.36(7)	0.18(8)	0.27(6)	0.04(7)	0.27(7)	0.24(6)
ethene–ethyne	1.09(8)	1.01(9)	1.03(8)	1.04(8)	1.09(6)	1.13(8)
ethene dimer	1.00(9)	1.17(8)	1.13(7)	1.20(10)	1.23(7)	1.37(9)
ethyne dimer	1.27(7)	1.28(9)	1.22(6)	1.23(7)	1.25(6)	1.20(8)

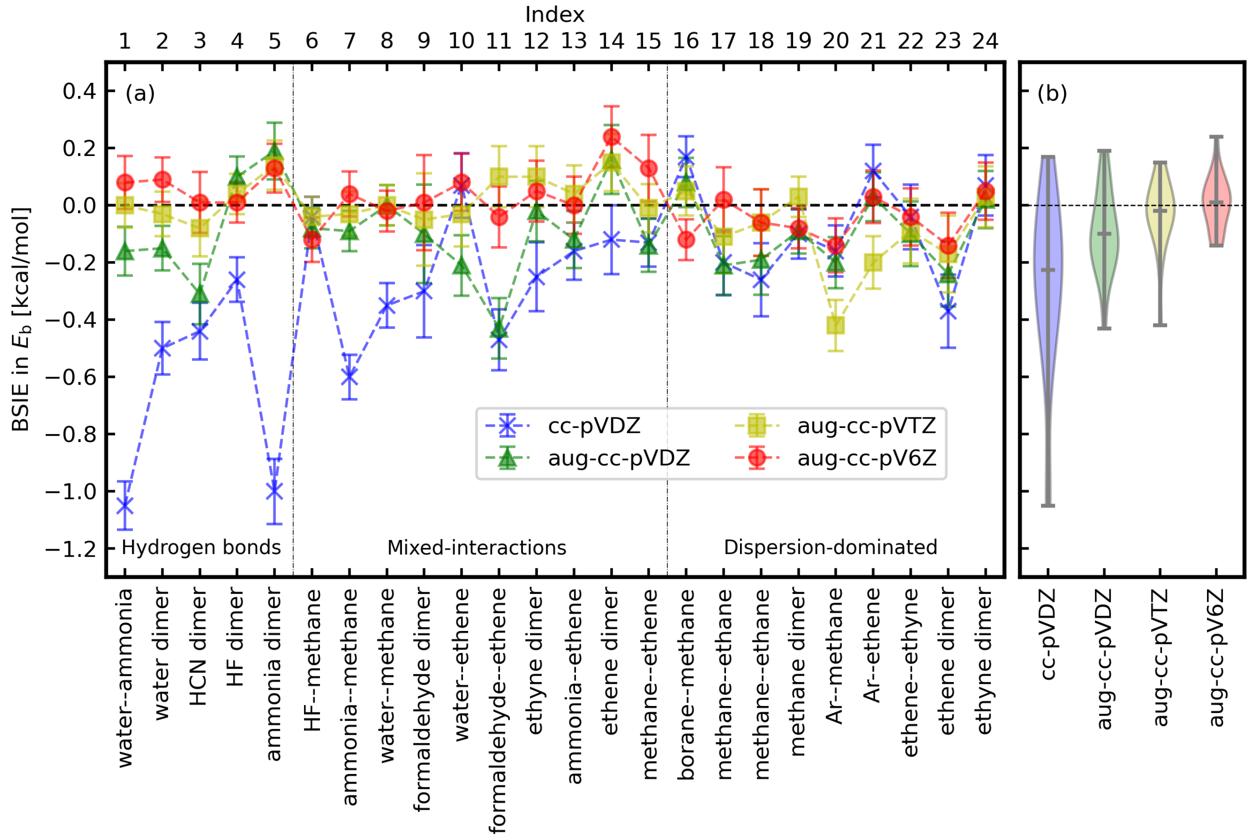

Figure S1: (a) BSIEs in the binding energies of the A24 set, estimated from DMC calculations with QMCPACK, where the reference values are those obtained with PW basis (i.e., the CBS limit). The error bars represent 1σ. (b) The violin plots for the obtained BSIEs. In panel (b), the mean values of the obtained binding energies for computing the distribution densities.

2 Supporting Results

Table SII: The total energies (E^{tot}) and variances (σ^2) of the wavefunctions for the water–ammonia dimer computed using VMC.

Basis	$E^{\mathrm{tot}}_{\mathrm{VMC}}$ (Ha)	σ^2_{VMC} (Ha^2)
VDZ	-23.377(1)	0.568(5)
VTZ	-23.399(1)	0.470(2)
VQZ	-23.400(1)	0.469(4)
V5Z	-23.398(1)	0.460(3)
V6Z	-23.403(1)	0.473(7)

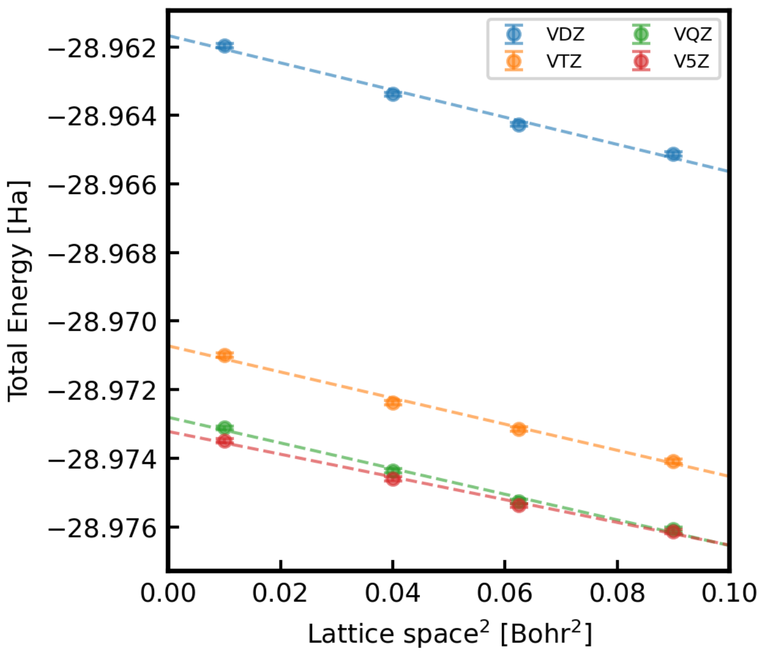

Figure S2: LRDMC energies of water–ammonia dimer with respect to the lattice space for various basis sets.

Table SIII: The binding energies (in kcal/mol) of the molecule contained in the A24-set, as obtained from FN-DMC evaluations using TurboRVB with the ccecp-cc-pVnZ (VnZ) basis set family (n=D, T, Q, 5, 6) without CP correction.

Label	VDZ	VTZ	VQZ	V5Z	V6Z
water–ammonia	-7.63(7)	-7.13(6)	-6.83(7)	-6.76(7)	-6.68(7)
water dimer	-5.73(7)	-5.47(7)	-5.29(7)	-5.15(7)	-5.19(7)
HCN dimer	-5.56(7)	-5.16(7)	-5.16(7)	-5.09(8)	-5.22(7)
HF dimer	-4.91(7)	-4.79(7)	-4.64(7)	-4.64(7)	-4.77(7)
ammonia dimer	-4.43(7)	-3.60(7)	-3.18(7)	-3.22(7)	-3.12(7)
HF–methane	-1.62(7)	-1.59(6)	-1.59(7)	-1.63(7)	-1.59(6)
ammonia–methane	-1.31(7)	-1.03(7)	-0.81(7)	-0.76(6)	-0.76(7)
water–methane	-0.94(8)	-0.57(7)	-0.86(6)	-0.62(6)	-0.65(7)
formaldehyde dimer	-4.78(6)	-4.79(8)	-4.63(7)	-4.56(9)	-4.60(9)
water–ethene	-2.69(8)	-2.79(7)	-2.64(6)	-2.71(6)	-2.65(7)
formaldehyde–ethene	-1.89(8)	-1.88(7)	-1.64(7)	-1.63(8)	-1.58(8)
ethyne dimer	-1.78(7)	-1.52(6)	-1.58(6)	-1.59(7)	-1.56(7)
ammonia–ethene	-1.43(6)	-1.39(7)	-1.46(7)	-1.42(8)	-1.38(7)
ethene dimer	-1.13(7)	-1.05(7)	-0.99(7)	-0.89(8)	-1.08(7)
methane–ethene	-0.55(8)	-0.47(6)	-0.47(8)	-0.55(6)	-0.48(7)
borane–methane	-1.16(7)	-1.35(7)	-1.49(6)	-1.23(7)	-1.33(6)
methane–ethane	-0.77(8)	-0.79(7)	-0.72(7)	-0.77(7)	-0.88(7)
methane–ethane	-0.64(7)	-0.43(7)	-0.64(6)	-0.68(6)	-0.51(6)
methane dimer	-0.46(7)	-0.55(7)	-0.48(6)	-0.38(7)	-0.47(6)
Ar–methane	-0.39(7)	-0.31(5)	-0.40(6)	-0.42(8)	-0.48(7)
Ar–ethene	-0.38(6)	-0.37(7)	-0.29(7)	-0.36(7)	-0.22(7)
ethene–ethyne	1.10(7)	1.08(6)	0.99(7)	0.91(7)	0.99(6)
ethene dimer	1.05(7)	0.95(7)	1.13(8)	1.16(7)	1.16(8)
ethyne dimer	1.33(7)	1.25(7)	1.17(7)	1.27(7)	1.45(6)

Table SIV: The binding energies (in kcal/mol) of the molecule contained in the A24-set, as obtained from FN-DMC evaluations using TurboRVB with the ccecp-aug-cc-pVnZ (aVnZ) basis set family (n=D, T, Q, 5, 6) without CP correction.

	aVDZ	aVTZ	aVQZ	aV5Z	aV6Z
water–ammonia	-6.73(7)	-6.61(6)	-6.65(6)	-6.64(6)	-6.75(7)
water dimer	-5.20(6)	-5.12(7)	-5.08(7)	-5.19(6)	-5.10(8)
HCN dimer	-5.54(7)	-5.09(7)	-5.11(7)	-5.03(8)	-5.09(7)
HF dimer	-4.70(7)	-4.56(7)	-4.62(8)	-4.78(7)	-4.74(7)
ammonia dimer	-3.19(6)	-3.12(7)	-3.04(6)	-3.18(7)	-3.10(6)
HF–methane	-1.90(7)	-1.72(7)	-1.65(7)	-1.53(7)	-1.64(7)
ammonia–methane	-0.69(7)	-0.71(7)	-0.70(7)	-0.68(6)	-0.80(7)
water–methane	-0.77(7)	-0.67(6)	-0.65(7)	-0.66(7)	-0.58(6)
formaldehyde dimer	-4.57(7)	-4.71(7)	-4.72(9)	-4.64(8)	-4.42(9)
water–ethene	-2.64(7)	-2.68(8)	-2.53(7)	-2.68(8)	-2.50(10)
formaldehyde–ethene	-1.81(7)	-1.65(7)	-1.70(8)	-1.43(9)	-1.71(10)
ethyne dimer	-1.59(7)	-1.71(6)	-1.61(7)	-1.58(7)	-1.44(7)
ammonia–ethene	-1.51(6)	-1.57(6)	-1.33(6)	-1.27(7)	-1.38(6)
ethene dimer	-1.19(6)	-1.04(7)	-0.96(8)	-1.05(9)	-0.97(9)
methane–ethene	-0.53(7)	-0.51(7)	-0.36(7)	-0.49(7)	-0.56(6)
borane–methane	-1.44(6)	-1.36(7)	-1.45(7)	-1.30(5)	-1.46(7)
methane–ethane	-0.93(7)	-0.83(6)	-0.78(8)	-0.71(8)	-0.65(9)
methane–ethane	-0.86(7)	-0.48(8)	-0.49(7)	-0.58(7)	-0.57(8)
methane dimer	-0.59(7)	-0.54(7)	-0.51(7)	-0.51(7)	-0.58(6)
Ar–methane	-0.35(7)	-0.44(7)	-0.45(7)	-0.33(7)	-0.36(8)
Ar–ethene	-0.40(7)	-0.29(7)	-0.31(7)	-0.34(8)	-0.24(7)
ethene–ethyne	1.03(8)	1.02(7)	0.98(8)	0.84(8)	1.04(9)
ethene dimer	0.97(7)	1.14(7)	1.06(8)	1.16(7)	1.04(8)
ethyne dimer	1.34(7)	1.27(7)	1.17(6)	1.28(7)	1.32(8)

Table SV: The binding energies (in kcal/mol) of ammonia dimer with LDA-PZ and B3LYP nodal surfaces, as obtained from FN-DMC evaluations using TurboRVB with the ccecp-cc-pVnZ (VnZ; n=D, T) and ccecp-aug-cc-pVnZ (aVnZ; n=D, T, Q, 5, 6) basis set families using the setup described in the main tex. $\Delta = E_{\mathrm{b}}^{\mathrm{LDA-PZ}} - E_{\mathrm{b}}^{\mathrm{B3LYP}}$.

	$E_{\mathrm{b}}^{\mathrm{LDA-PZ}}$	$E_{\mathrm{b}}^{\mathrm{B3LYP}}$	Δ
VDZ	-4.43(7)	-4.46(6)	0.03(9)
VTZ	-3.60(7)	-3.86(7)	0.26(10)
aVDZ	-3.19(6)	-3.10(7)	-0.09(9)
aVTZ	-3.12(7)	-3.21(7)	0.09(10)
aVQZ	-3.04(6)	-3.02(8)	-0.02(10)
aV5Z	-3.18(7)	-3.09(6)	-0.09(9)
aV6Z	-3.10(6)	-3.13(7)	0.03(9)

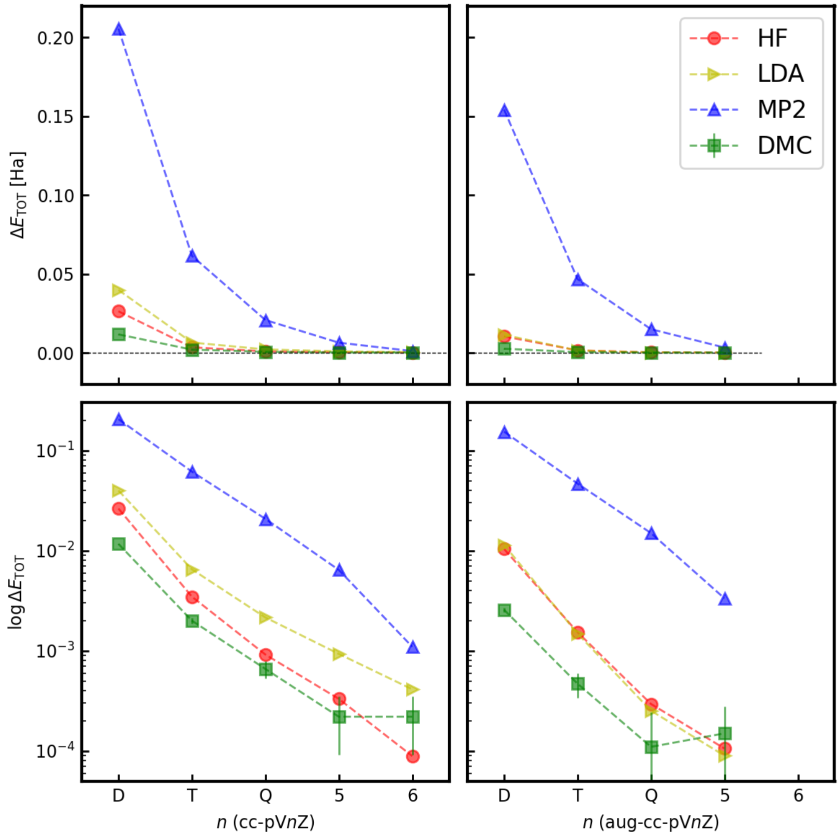

Figure S3: Total Energy of the water–ammonia dimer in the A24 data set with respect to the energy obtained with the aug-cc-pV6Z basis set.

Table SVI: The binding energies (in kcal/mol) of ammonia dimer with LDA-PZ, PBE, ωB97M-V, PBE0, B3LYP and HF nodal surfaces, as obtained from FN-DMC evaluations using QMCPACK with the ccecp-cc-pVnZ (VnZ; n=D, T, Q, 5, 6) and ccecp-aug-cc-pVnZ (aVnZ; n=D, T, Q, 5, 6) basis set families using the setup described in Sec. 1 for a τ = 0.003 au timestep.

	$E_{\rm b}^{\rm LDA\text{-}PZ}$	$E_{\rm b}^{\rm PBE}$	$E_{\rm b}^{\omega{\rm B97M\text{-}V}}$	$E_{\rm b}^{\rm PBE0}$	$E_{\rm b}^{\rm B3LYP}$	$E_{\rm b}^{\rm HF}$
VDZ	-4.29(5)	-4.34(5)	-4.36(6)	-4.15(6)	-4.37(6)	-4.10(6)
VTZ	-3.66(7)	-3.79(5)	-3.66(5)	-3.59(5)	-3.80(5)	-3.67(4)
VQZ	-3.31(5)	-3.23(4)	-3.42(5)	-3.42(4)	-3.38(5)	-3.38(6)
V5Z	-3.42(7)	-3.37(5)	-3.30(4)	-3.27(5)	-3.27(4)	-3.31(5)
V6Z	-3.25(3)	-3.20(5)	-3.30(4)	-3.16(4)	-3.32(4)	-3.21(7)
aVDZ	-3.27(6)	-3.16(5)	-3.22(5)	-3.14(7)	-3.25(6)	-3.24(5)
aVTZ	-3.24(5)	-3.18(4)	-3.18(6)	-3.17(4)	-3.18(5)	-3.22(5)
aVQZ	-3.23(4)	-3.13(5)	-3.30(5)	-3.24(4)	-3.21(4)	-3.20(5)
aV5Z	-3.17(4)	-3.27(4)	-3.25(5)	-3.13(4)	-3.23(5)	-3.17(6)
aV6Z	-3.17(5)	-3.18(5)	-3.14(4)	-3.15(4)	-3.10(5)	-3.15(4)

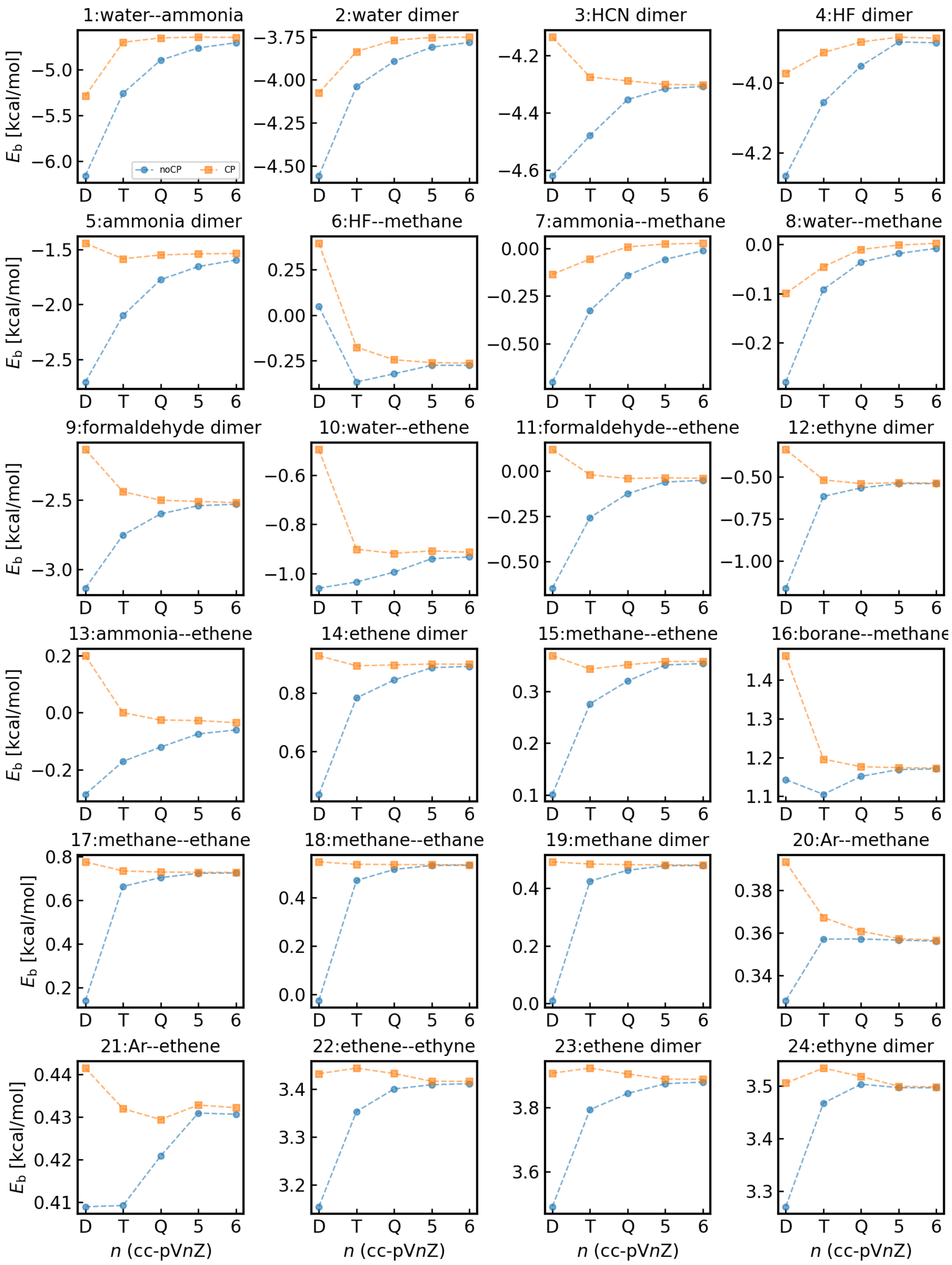

Figure S4: The binding energies of A24 data set computed by HF with cc-pVnZ basis sets (n=D,T,Q,5,6). The CP and noCP represent 'with the counterpoise correction' and 'without the counterpoise correction', respectively.

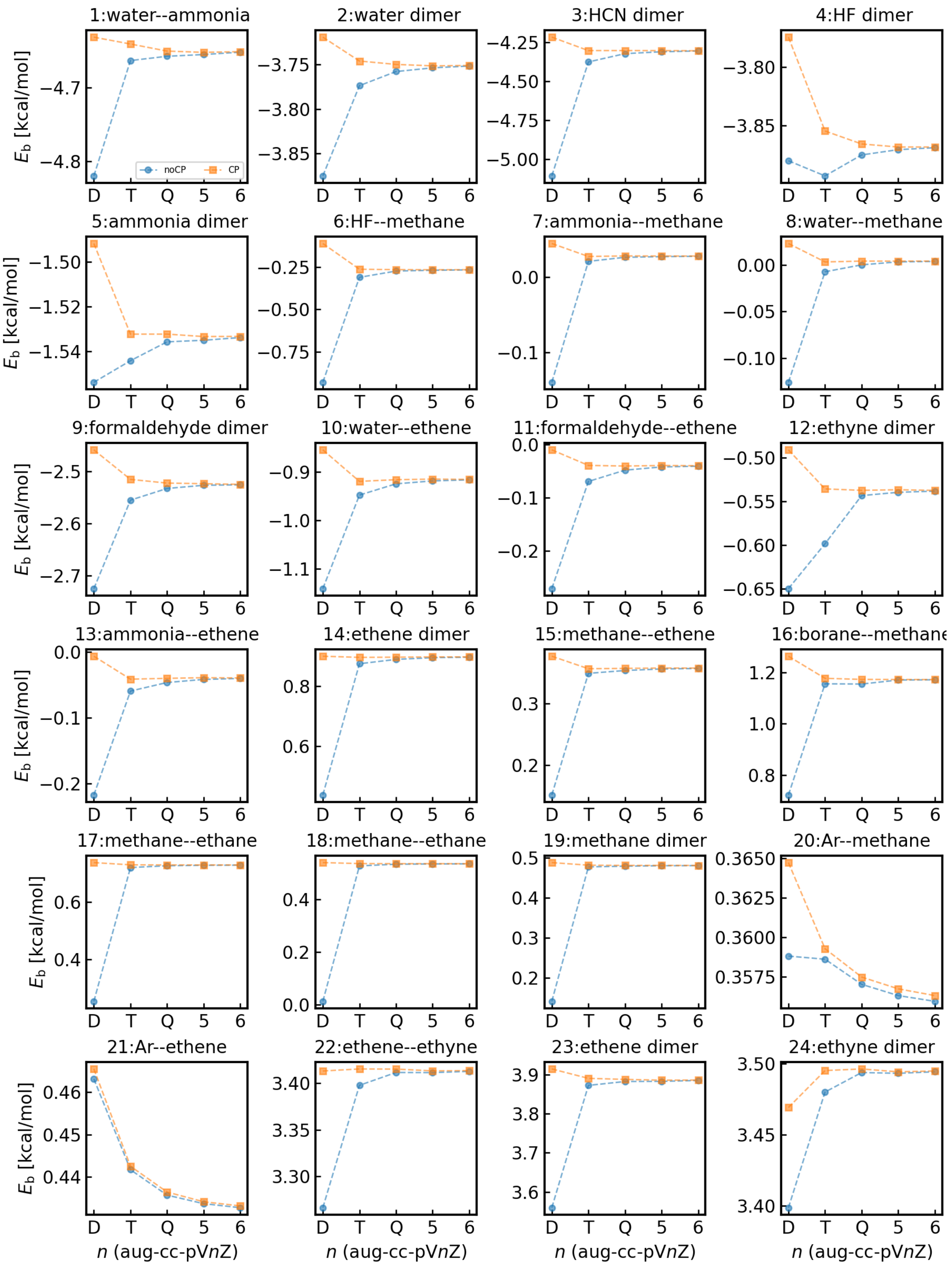

Figure S5: The binding energies of A24 data set computed by HF with aug-cc-pVnZ basis sets (n=D,T,Q,5,6). The CP and noCP represent 'with the counterpoise correction' and 'without the counterpoise correction', respectively.

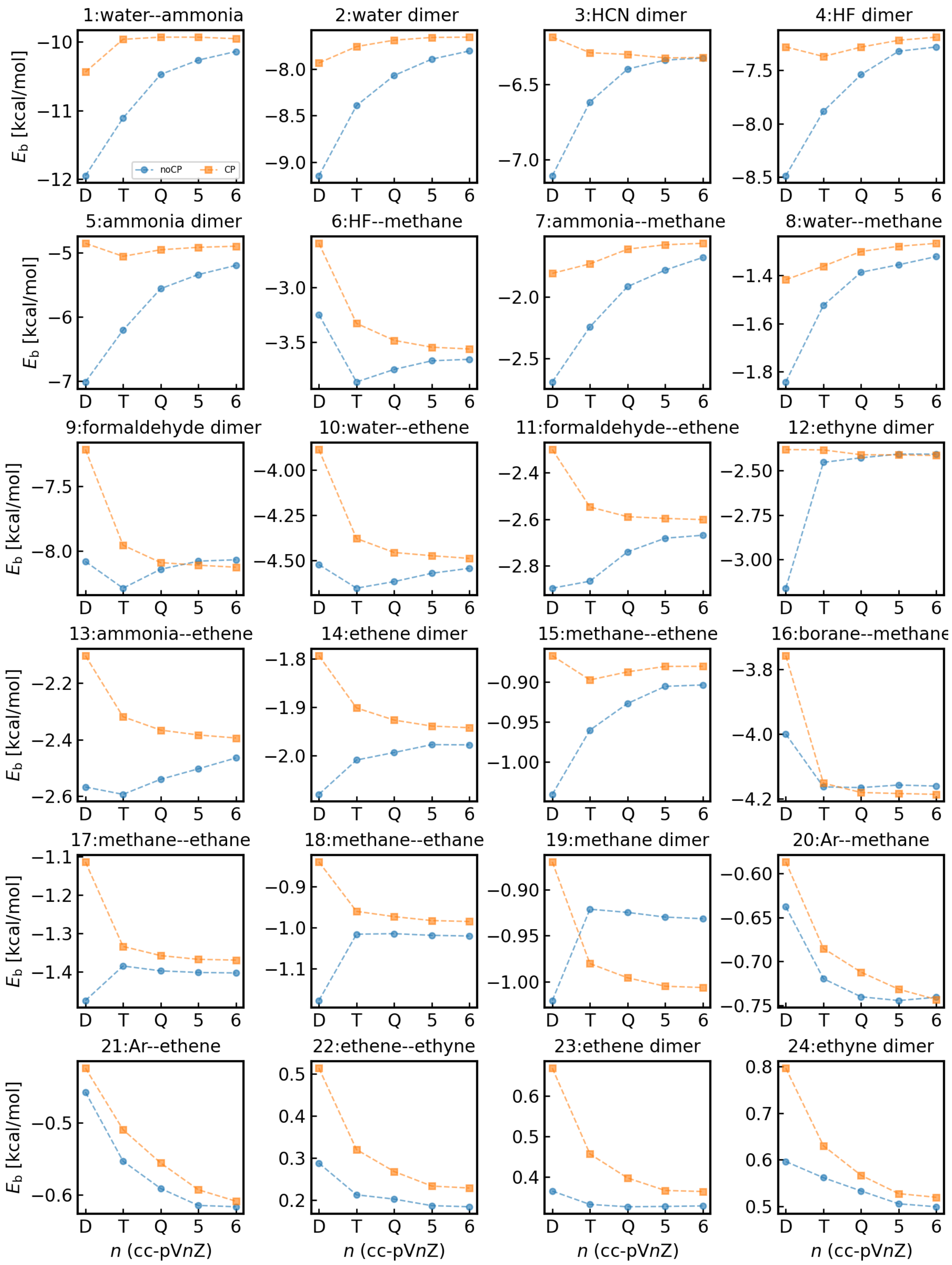

Figure S6: The binding energies of A24 data set computed by LDA with cc-pVnZ basis sets (n=D,T,Q,5,6). The CP and noCP represent 'with the counterpoise correction' and 'without the counterpoise correction', respectively.

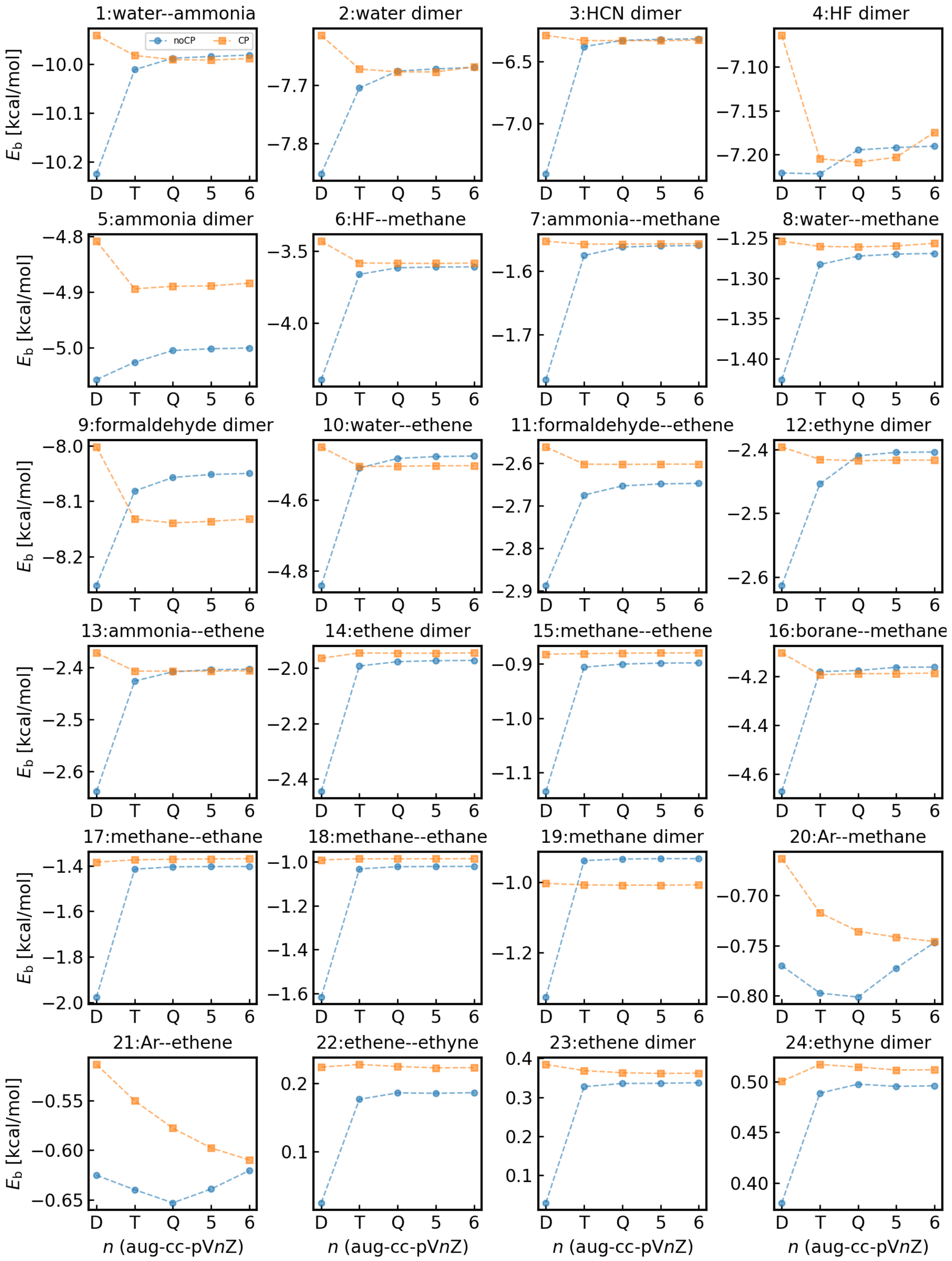

Figure S7: The binding energies of A24 data set computed by LDA with aug-cc-pVnZ basis sets (n=D,T,Q,5,6). The CP and noCP represent 'with the counterpoise correction' and 'without the counterpoise correction', respectively.

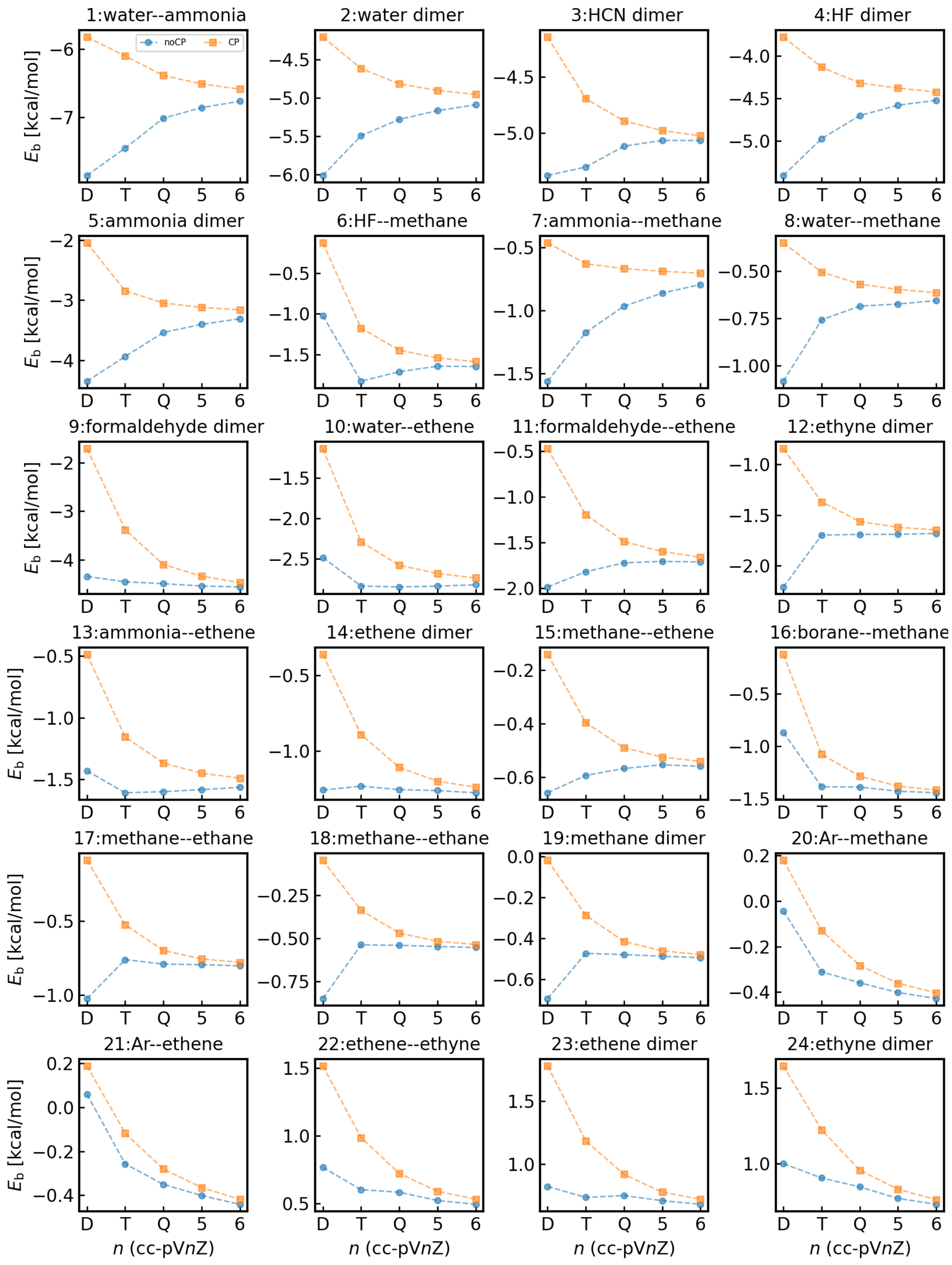

Figure S8: The binding energies of A24 data set computed by MP2 with cc-pVnZ basis sets (n=D,T,Q,5,6). The CP and noCP represent 'with the counterpoise correction' and 'without the counterpoise correction', respectively.

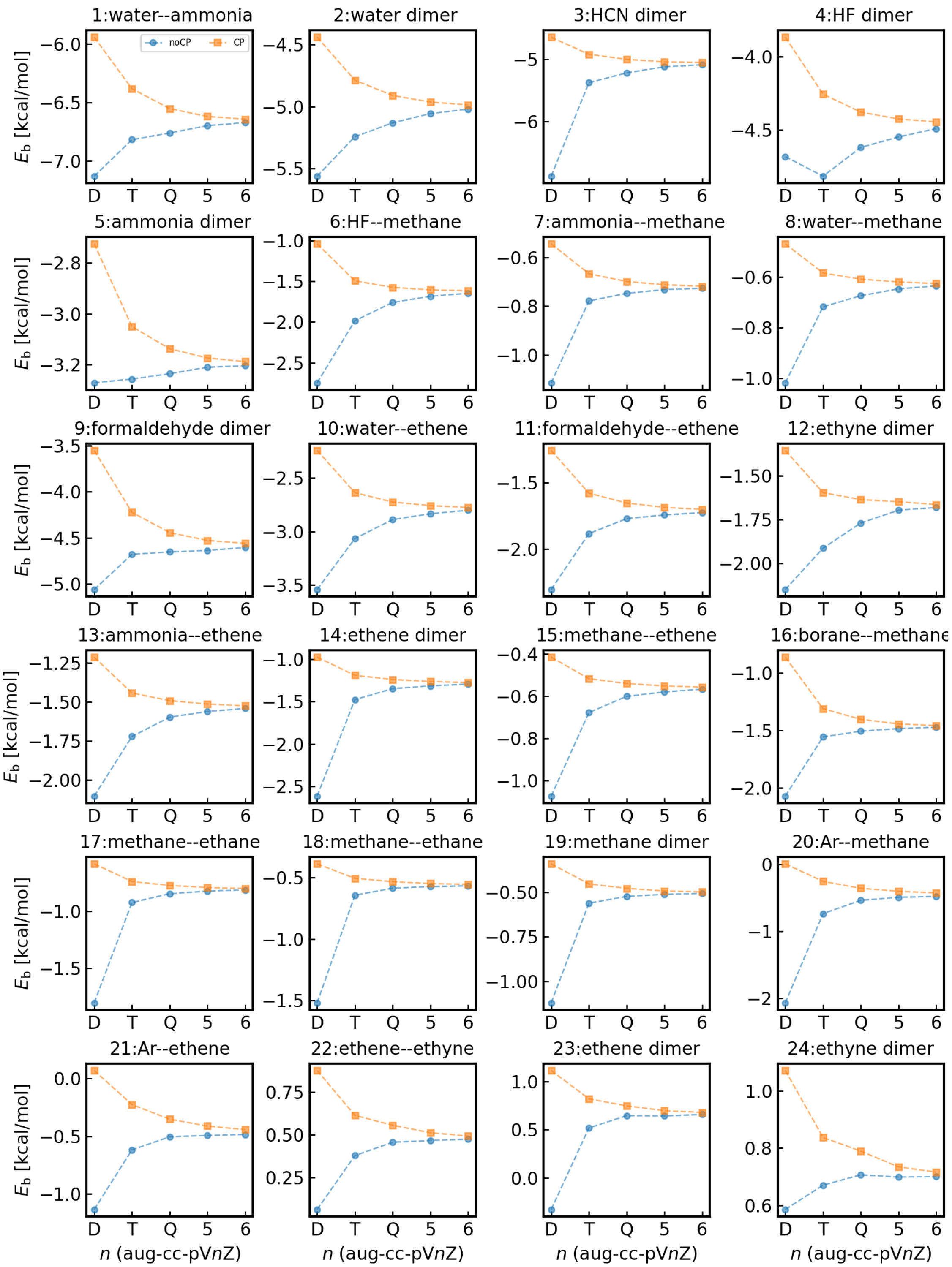

Figure S9: The binding energies of A24 data set computed by MP2 with aug-cc-pVnZ basis sets (n=D,T,Q,5,6). The CP and noCP represent 'with the counterpoise correction' and 'without the counterpoise correction', respectively.

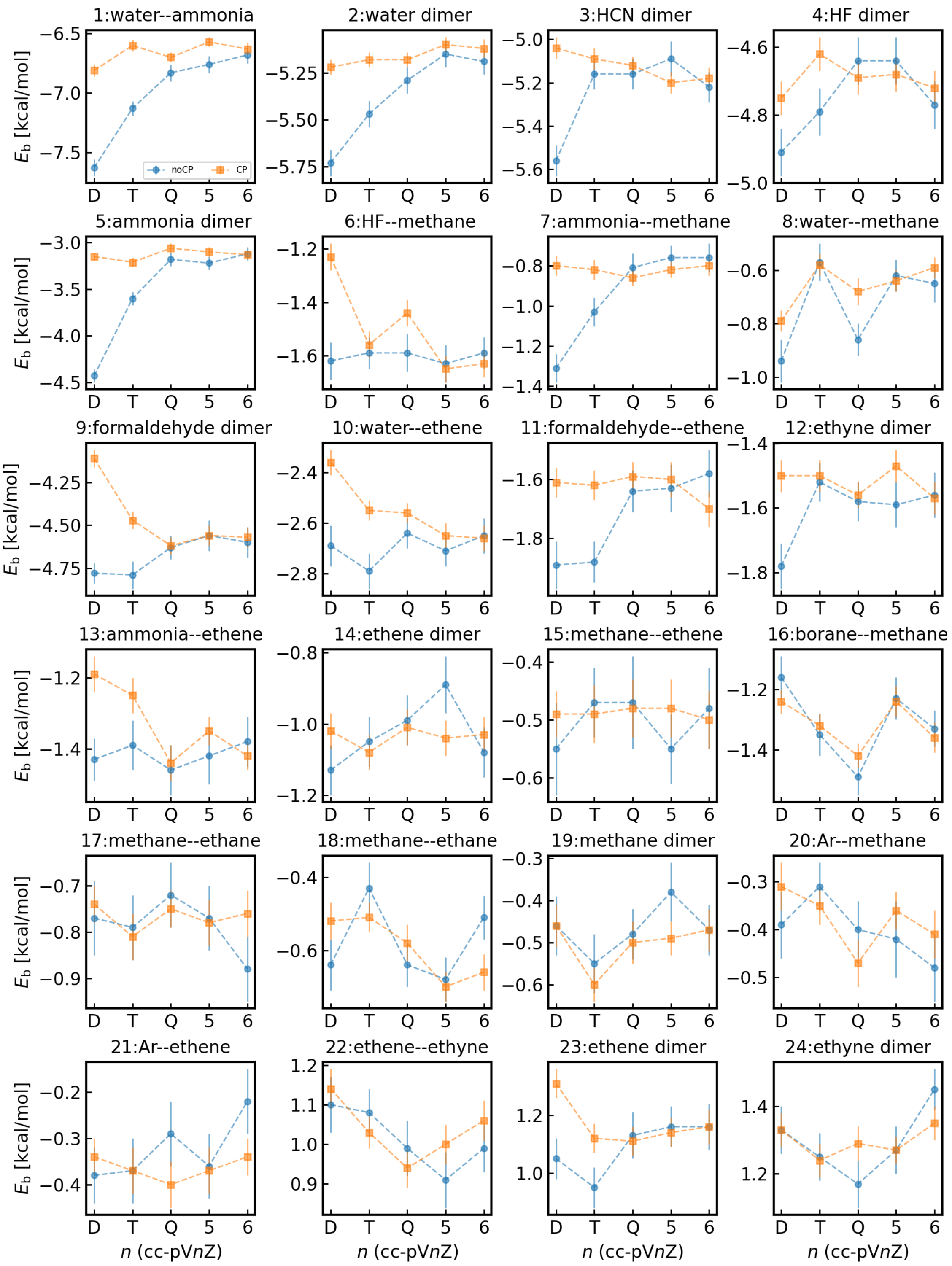

Figure S10: The binding energies of A24 data set computed by LRDMC with cc-pVnZ basis sets (n=D,T,Q,5,6). The CP and noCP represent 'with the counterpoise correction' and 'without the counterpoise correction', respectively.

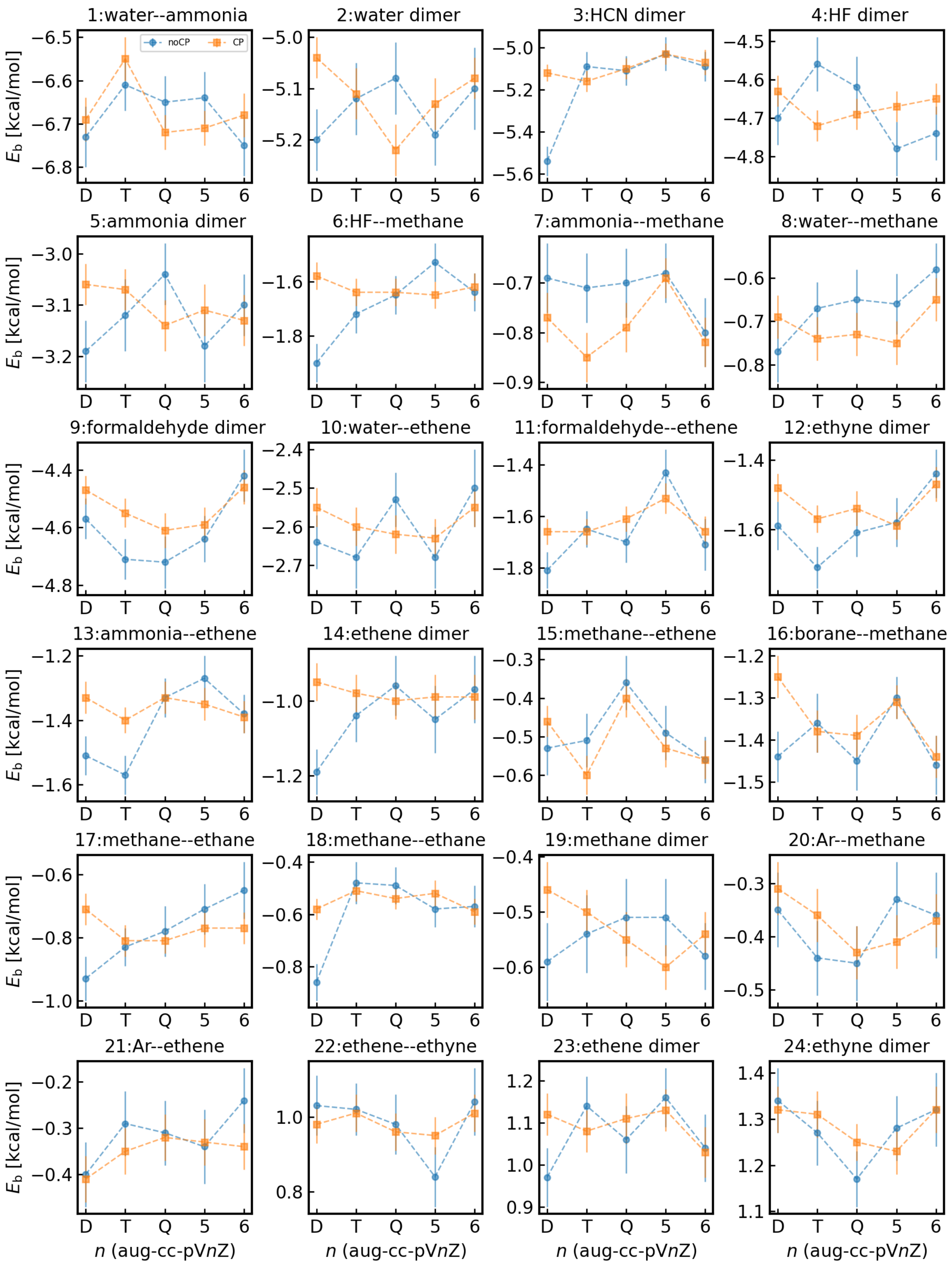

Figure S11: The binding energies of A24 data set computed by LRDMC with aug-cc-pVnZ basis sets (n=D,T,Q,5,6). The CP and noCP represent 'with the counterpoise correction' and 'without the counterpoise correction', respectively.

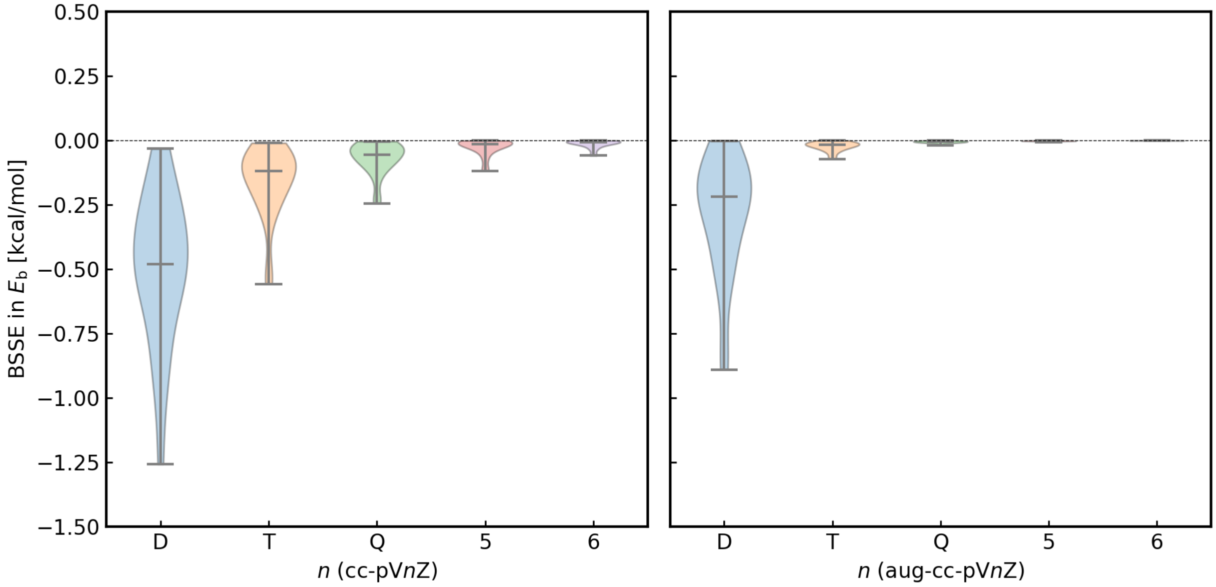

Figure S12: The Violin plots of BSSEs in the binding energies of A24 data set computed by HF with (left) cc-pVnZ basis sets (n=D,T,Q,5,6) and (right) with aug-cc-pVnZ basis sets (n=D,T,Q,5,6).

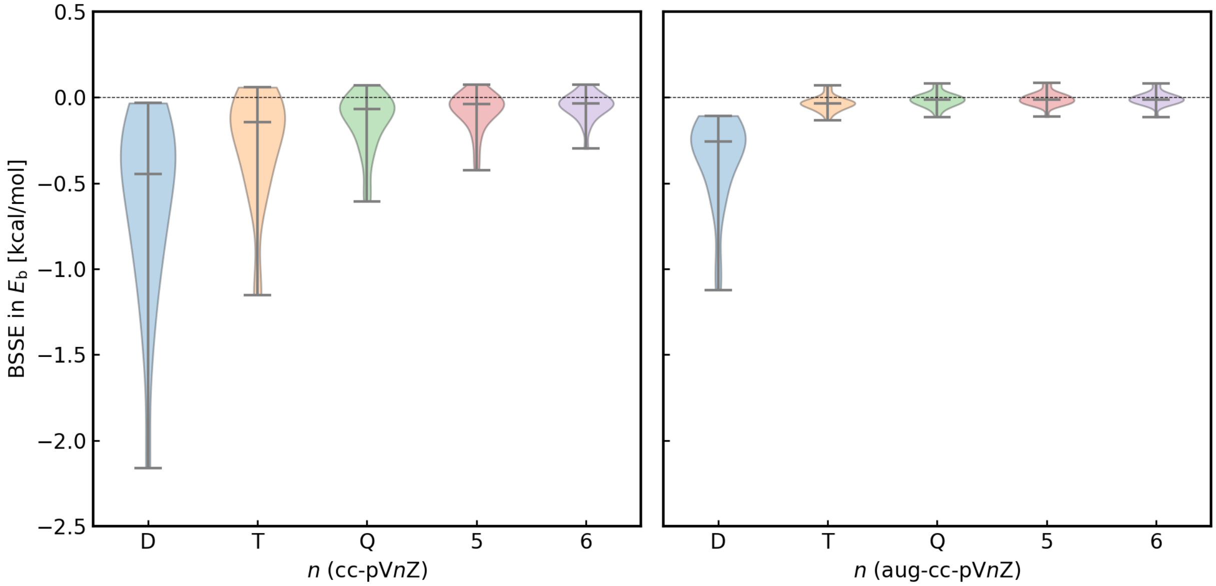

Figure S13: The Violin plots of BSSEs in the binding energies of A24 data set computed by LDA with (left) cc-pVnZ basis sets (n=D,T,Q,5,6) and (right) with aug-cc-pVnZ basis sets (n=D,T,Q,5,6).

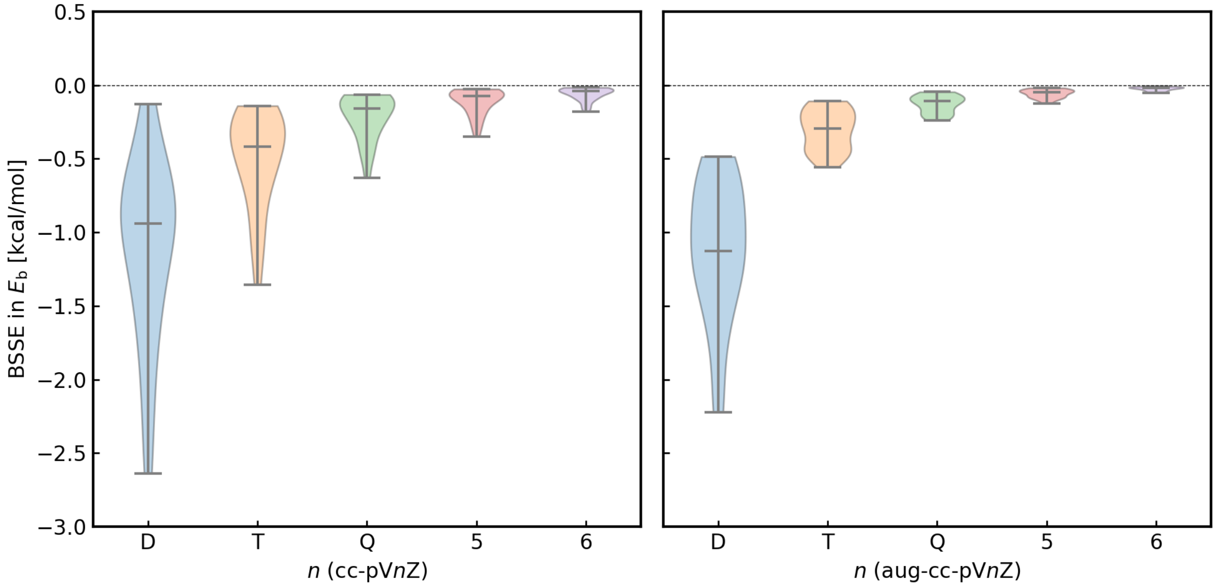

Figure S14: The Violin plots of BSSEs in the binding energies of A24 data set computed by MP2 with (left) cc-pVnZ basis sets (n=D,T,Q,5,6) and (right) with aug-cc-pVnZ basis sets (n=D,T,Q,5,6).

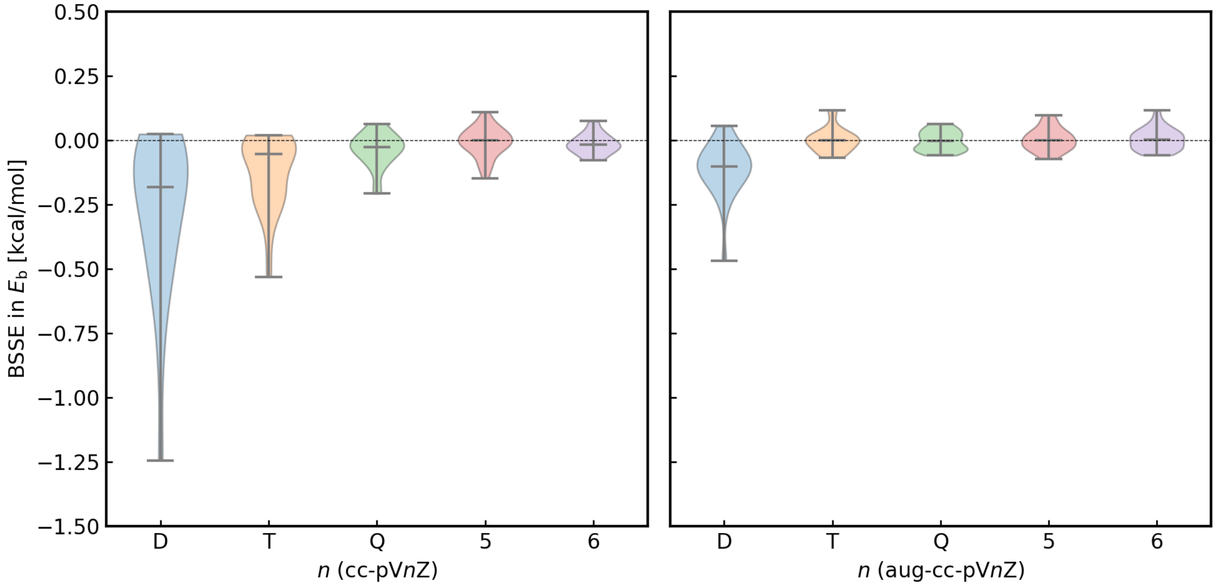

Figure S15: The Violin plots of BSSEs in the binding energies of A24 data set computed by LRDMC with (left) cc-pVnZ basis sets (n=D,T,Q,5,6) and (right) with aug-cc-pVnZ basis sets (n=D,T,Q,5,6).

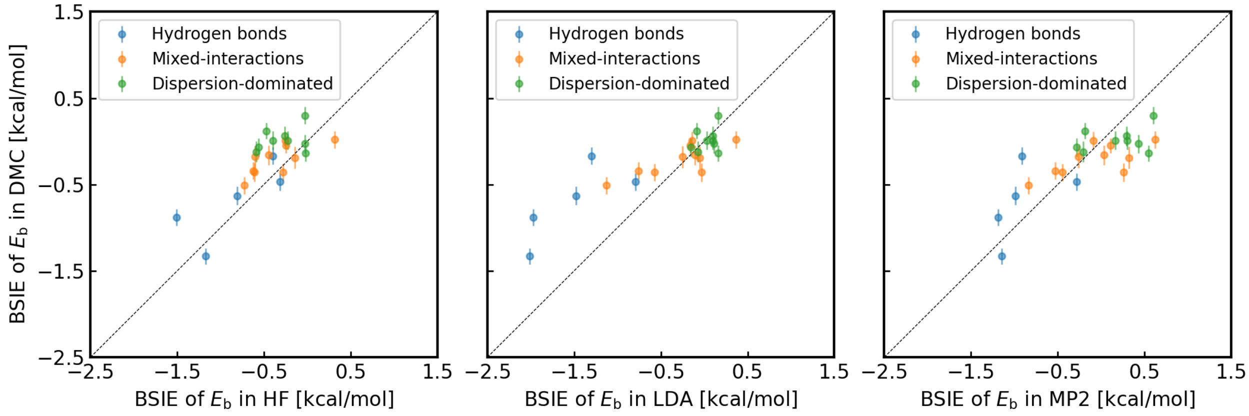

Figure S16: Comparison of BSIEs in the binding energy calculations with cc-pVDZ basis set by DMC with those by HF, LDA, and MP2.

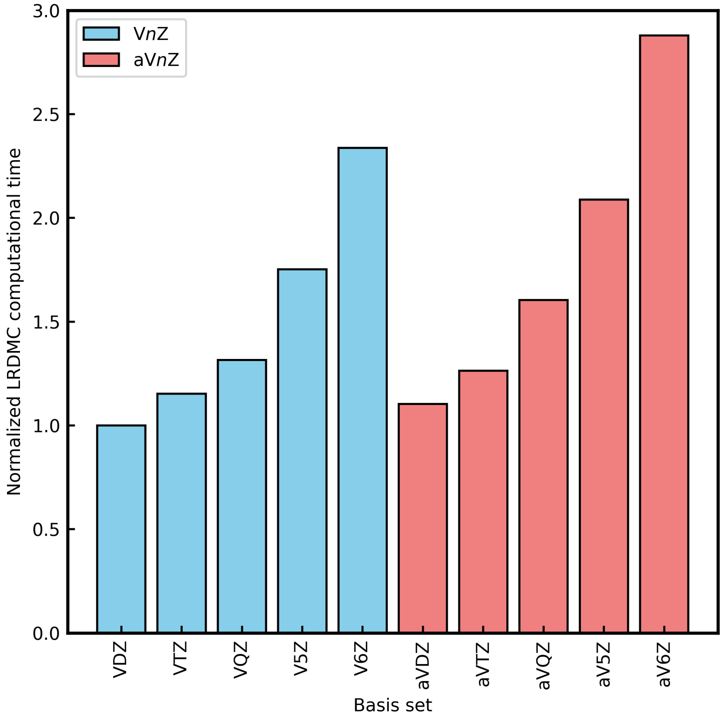

Figure S17: Normalized LRDMC computational time of the water dimer with respect to the basis set, as obtained from FN-DMC evaluations using TurboRVB. They were measured on the RIKEN supercomputer Fugaku using 32 nodes (i.e., 1536 cores with 1536 MPI processes). The lattice discretization was 0.2 Bohr.

[1] Kim, J.; Baczewski, A. D.; Beaudet, T. D.; Benali, A.; Bennett, M. C.; Berrill, M. A.; Blunt, N. S.; Borda, E. J. L.; Casula, M.; Ceperley, D. M.; Chiesa, S.; Clark, B. K.; Clay, R. C.; Delaney, K. T.; Dewing, M.; Esler, K. P.; Hao, H.; Heinonen, O.; Kent, P. R. C.; Krogel, J. T.; Kylänpää, I.; Li, Y. W.; Lopez, M. G.; Luo, Y.; Malone, F. D.; Martin, R. M.; Mathuriya, A.; McMinis, J.; Melton, C. A.; Mitas, L.; Morales, M. A.; Neuscamman, E.; Parker, W. D.; Flores, S. D. P.; Romero, N. A.; Rubenstein, B. M.; Shea, J. A. R.; Shin, H.; Shulenburger, L.; Tillack, A. F.; Townsend, J. P.; Tubman, N. M.; Goetz, B. V. D.; Vincent, J. E.; Yang, D. C.; Yang, Y.; Zhang, S.; Zhao, L. QMCPACK: an open sourceab initioquantum Monte Carlo package for the electronic structure of atoms, molecules and solids. *J. Phys. Condens. Matter* **2018**, *30*, 195901.

[2] Kent, P. R.; Annaberdiyev, A.; Benali, A.; Bennett, M. C.; Borda, E. J. L.; Doak, P.; Hao, H.; Jordan, K. D.; Krogel, J. T.; Kylänpaä, I.; Lee, J.; Luo, Y.; Malone, F. D.; Melton, C. A.; Mitas, L.; Morales, M. A.; Neuscamman, E.; Reboredo, F. A.; Rubenstein, B.; Saritas, K.; Upadhyay, S.; Wang, G.; Zhang, S.; Zhao, L. QMCPACK: Advances in the development, efficiency, and application of auxiliary field and real-space variational and diffusion quantum Monte Carlo. *J. Chem. Phys.* **2020**, *152*.

[3] Bennett, M. C.; Melton, C. A.; Annaberdiyev, A.; Wang, G.; Shulenburger, L.; Mitas, L. A new generation of effective core potentials for correlated calculations. *J. Chem. Phys* **2017**, *147*, 224106.

[4] Bennett, M. C.; Wang, G.; Annaberdiyev, A.; Melton, C. A.; Shulenburger, L.; Mitas, L. A new generation of effective core potentials from correlated calculations: 2nd row elements. *J. Chem. Phys.* **2018**, *149*, 104108.

[5] Perdew, J. P.; Zunger, A. Self-interaction correction to density-functional approximations for many-electron systems. *Phys. Rev. B* **1981**, *23*, 5048.

[6] Sun, Q.; Berkelbach, T. C.; Blunt, N. S.; Booth, G. H.; Guo, S.; Li, Z.; Liu, J.; McClain, J. D.; Sayfutyarova, E. R.; Sharma, S., et al. PySCF: the Python-based simulations of chemistry framework. *Wiley Interdiscip. Rev. Comput. Mol. Sci.* **2018**, *8*, e1340.

[7] Sun, Q.; Zhang, X.; Banerjee, S.; Bao, P.; Barbry, M.; Blunt, N. S.; Bogdanov, N. A.; Booth, G. H.; Chen, J.; Cui, Z. H.; Eriksen, J. J.; Gao, Y.; Guo, S.; Hermann, J.; Hermes, M. R.; Koh, K.; Koval, P.; Lehtola, S.; Li, Z.; Liu, J.; Mardirossian, N.; McClain, J. D.; Motta, M.; Mussard, B.; Pham, H. Q.; Pulkin, A.; Purwanto, W.; Robinson, P. J.; Ronca, E.; Sayfutyarova, E. R.; Scheurer, M.; Schurkus, H. F.; Smith, J. E.; Sun, C.; Sun, S. N.; Upadhyay, S.; Wagner, L. K.; Wang, X.; White, A.; Whitfield, J. D.; Williamson, M. J.; Wouters, S.; Yang, J.; Yu, J. M.; Zhu, T.; Berkelbach, T. C.; Sharma, S.; Sokolov, A. Y.; Chan, G. K. L. Recent developments in the PySCF program package. *J. Chem. Phys.* **2020**, *153*, 024109.

[8] Giannozzi, P.; Baroni, S.; Bonini, N.; Calandra, M.; Car, R.; Cavazzoni, C.; Ceresoli, D.; Chiarotti, G. L.; Cococcioni, M.; Dabo, I., et al. QUANTUM ESPRESSO: a modular and open-source software project for quantum simulations of materials. *J. Phys. Condens. Matter.* **2009**, *21*, 395502.

[9] Alfè, D.; Gillan, M. J. Efficient localized basis set for quantum Monte Carlo calculations on condensed matter. *Phys. Rev. B* **2004**, *70*, 161101.

[10] Casula, M. Beyond the Locality Approximation in the Standard Diffusion Monte Carlo Method. *Phys. Rev. B* **2006**, *74*, 161102.

[11] Zen, A.; Sorella, S.; Gillan, M. J.; Michaelides, A.; Alfè, D. Boosting the accuracy and speed of quantum Monte Carlo: Size consistency and time step. *Phys. Rev. B* **2016**, *93*, 241118(R).
